# Whole Genomes of Chandipura Virus Isolates and Comparative Analysis with Other Rhabdoviruses

**DOI:** 10.1371/journal.pone.0030315

**Published:** 2012-01-17

**Authors:** Sarah S. Cherian, Rashmi S. Gunjikar, Arpita Banerjee, Satyendra Kumar, Vidya A. Arankalle

**Affiliations:** National Institute of Virology, Pashan, Pune, Maharashtra, India; Virginia Polytechnic Institute and State University, United States of America

## Abstract

The Chandipura virus (CHPV) belonging to the *Vesiculovirus* genus and *Rhabdoviridae* family, has recently been associated with a number of encephalitis epidemics, with high mortality in children, in different parts of India. No full length genome sequences of CHPV isolates were available in GenBank and little is known about the molecular markers for pathogenesis. In the present study, we provide the complete genomic sequences of four isolates from epidemics during 2003–2007. These sequences along with the deduced sequence of the prototype isolate of 1965 were analysed using phylogeny, motif search, homology modeling and epitope prediction methods. Comparison with other rhaboviruses was also done for functional extrapolations. All CHPV isolates clustered with the Isfahan virus and maintained several functional motifs of other rhabdoviruses. A notable difference with the prototype vesiculovirus, Vesicular Stomatitis Virus was in the L-domain flanking sequences of the M protein that are known to be crucial for interaction with host proteins. With respect to the prototype isolate, significant additional mutations were acquired in the 2003–2007 isolates. Several mutations in G mapped onto probable antigenic sites. A mutation in N mapped onto regions crucial for N-N interaction and a putative T-cell epitope. A mutation in the Casein kinase II phosphorylation site in P may attribute to increased rates of phosphorylation. Gene junction comparison revealed changes in the M-G junction of all the epidemic isolates that may have implications on read-through and gene transcription levels. The study can form the basis for further experimental verification and provide additional insights into the virulence determinants of the CHPV.

## Introduction

Chandipura Virus (CHPV), a member of the family Rhabdoviridae and genus Vesiculovirus was first isolated in 1965 from two febrile cases during an outbreak of Dengue and Chikungunya in Nagpur, Maharashtra state, India [],[2]. In recent years, it has been associated with a number of encephalitis epidemics in different states of India; Andhra Pradesh (AP) in 2003 [3] and 2007, Gujarat in 2004 [4], and Maharashtra in 2007 [5] and 2009. Further, the virus was also shown to be responsible for sporadic encephalitis in children from AP [6]. Although, the virus closely resembles the prototype Vesiculovirus, Vesicular Stomatitis Virus (VSV), it could be readily distinguished by its ability to infect humans [7]. The CHPV has also been isolated in Nigeria from hedgehogs [8] and in Sri Lanka from macaques [9]. Sandflies are considered as the vectors for this pathogen [10],[11] while antibodies against the virus have been detected in a wide range of vertebrate animals [12]. Cells of insect origin and vertebrate animals were found to be susceptible to virus replication [13].

The viral genome of CHPV consists of a linear, single stranded negative sense RNA molecule of approximately 11,120 base pairs. A 49 nucleotide leader RNA is transcribed from the 3′ genomic terminus, which is non-translated, uncapped, and non-polyadenylated. Transcription of viral genes occurs in a sequential manner from a single promoter at the 3′ end of the genome resulting in a decreasing amount of each transcript in the order 3′-N-P-M-G-L-5′ similar to VSV [7],[14]. The five genes encode the respective proteins of different functionalities. The nucleocapsid (N) binds with nascent leader RNA and initiates encapsidation of the replication product concurrent to synthesis [15],[16]. The phosphoprotein (P) plays an important role in the transcription and replication of CHPV [17]–[19]. The matrix protein (M) represents an important component in the virus structure, assembly and budding and was shown to have the ability to inhibit gene expression [20]. The spike glycoprotein (G) is responsible for virus entry into cells and induction of neutralizing antibodies. The precise mechanism of CHPV fusion has not been elucidated so far. However, for VSV, it has been proposed that low pH induced conformational change in the G protein within the endosome subsequent to viral entry enables membrane fusion to release the core particle in two sequential steps into the host cytoplasm [21]. The large protein (L) is a subunit of the RNA polymerase complex. It retains the enzymatic activities of polymerase, capping, and polyadenylation. A 46 nucleotide trailer sequence at the 5′ end remains untranscribed [7].

The CHPV encephalitis epidemics have been consistently reported to be associated with a very high case fatality rate (CFR) ranging from 55–78% [3],[4]. The virus has thus emerged as an important pathogen. With the initial association of CHPV with the epidemics of encephalitis in children [3] the G, N and P genes of the AP virus isolates from the 2003 outbreak were compared with the 1965 prototype though the associations of the observed mutations to the pathogenesis remained to be fully determined [22]. No full-length sequences of CHPV isolates were available in GenBank. Further, compared to other *rhabodoviruses,* an in-depth genomics- and proteomics-based characterization of CHPV has not been reported.

The present study provides the complete genomic sequences of four isolates representing the major recent epidemics in AP, 2003 and 2007; Gujarat, 2004; and Maharashtra, 2007. We also deduced the full genome sequence of the 1965 prototype isolate from the available GenBank sequences [22],[23]. We carried out a sequence/structure-based characterization of the deduced protein sequences, genomic termini and the gene junctions of the isolates from the epidemics vis-à-vis the prototype isolate. Wherever appropriate, comparisons were performed with other members of the *Rhabdoviridae* family such as VSV, Isfahan virus (ISFV) (*genus: Vesiculovirus*), rabies virus (RABV) (*genus: Lyssavirus*) and Bovine Ephemeral fever virus (BEFV) (*genus: Ephemerovirus*) and also more divergent and newer rhabdoviruses such as the turbot rhabdovirus, Scopthalmus maximus rhabdovirus (SMRV). An attempt has also been made to understand the difference in pathogenicity between the 1965 virus and the recent CHPV virus isolates.

## Methods

### Virus And Rna Isolation

The clinical materials (throat swabs/serum) were obtained from infected children (between the ages of 4 and 16 years) from the CHPV epidemics during the period 2003 – 2007. The viruses were passaged twice in rhabdomyosarcoma (RD) cell line. The viral RNA was isolated using QIAamp Viral RNA Mini kit (QIAGEN, Valencia, CA) according to the manufacturer's instructions. Four CHPV isolates from AP (CIN0327, CIN0755), Gujarat (CIN0451) and Maharashtra (CIN0728) were chosen for full-length genome sequencing. The GenBank accession numbers of these four whole genome sequences are GU212856.1 (CIN0327), GU212857.1 (CIN0451), GU212858.1 (CIN0751) and GU190711.1 (CIN0755). The full genome sequence of the 1965 prototype isolate (CIN6514) was deduced from the available GenBank sequences having accession numbers AY614724, AF128868, J04350, AY614717 and AJ810083.

### Rt-Pcr And Sequencing

SuperScript™ II reverse transcriptase was used for the cDNA synthesis and Platinum Pfx DNA polymerase was used for the amplification according to the manufacturer's instructions (Invitrogen, CA). Amplified fragments were visualized by ethidium bromide agarose gel staining, extracted from the gels and sequenced directly using BigDye Terminator Cycle Sequencing kit (Applied Biosystems, CA).

For the designing of primers, the deduced nucleotide sequence of the 1965 isolate was used. The N, P, M and G genes were amplified in a single stretch and the L gene was amplified in four overlapping fragments.

The 3′ and 5′ end of all the isolates were amplified using primers LeF1 and NF7 and LF13 and TeR1 respectively. The primers LeF1 and TeR1 were used as described by Nichol & Holland, 1987. The amplified products were cloned into pGEM T easy vector (Promega, Madison, USA) and sequenced using T-7 and SP-6 promoter primers. Table S1 provides the list of primers used. As the isolates have undergone low levels of passage, the possibility of mutations being incorporated during cell culture adaptation, can be ruled out. The Genbank accession numbers of the isolates are: GU212858.1 (CIN0327), GU212856.1 (CIN0451), GU190711.1 (CIN0728) and GU212857.1 (CIN0751).

### Multiple Sequence Alignments And Phylogenetic Trees

**Figure 1:**
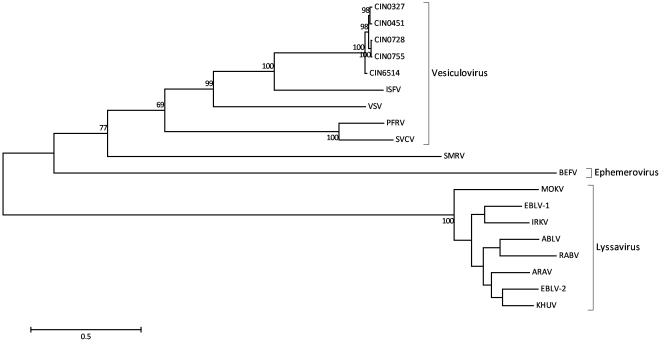
Phylogenetic tree of the whole genomes of the CHPV isolates and other rhabdoviruses using the Maximum likelihood method as implemented in MEGA. v.5.05. Bootstrap values greater than 60% are indicated at the appropriate nodes. Scale bar indicates number of nucleotide substitutions per site. Abbreviations: CHPV (Chandipura virus; acc nos. GU212856.1-58.1 [CIN0327, CIN0451, CIN0751], GU190711.1 [CIN0728], CIN6514 [deduced 1965 CHPV sequence]); ISFV (Isfahan virus; acc. no. AJ810084.2); VSV (vesicular stomatitis virus; acc. no. NC_001560.1); PFRV (pike fry rhabdovirus; acc. no. FJ872827.1); SVCV (spring viraemia of carp virus; acc. no. DQ097384.2); SMRV (Scopthalmus maximus rhabdovirus; acc. no. HQ003891.1); BEFV (Bovine Ephemeral fever virus; acc. no. AF234533.1); MOKV (Mokola virus; acc. no. NC_006429.1); EBLV-1 (European bat lyssavirus-1; acc. no. EU293112.1); IRKV (Irkut virus; acc. no. EF614260.1); ABLV (Australian bat lyssavirus; acc. no. AF418014.1); RABV (rabies virus; acc. no. GQ918139); ARAV (Aravan virus; acc. no. EF614259.1); EBLV-2 (European bat lyssavirus-2; acc. no. NC_009528.1); KHUV (Khujand virus; acc. no. EF614261.1).

Whole genome sequences were aligned using ClustalW as implemented in MEGA. v.5.05 [24]. Phylogenetic analyses of the whole genomes of CHPV along with other rhabdoviruses were carried out in MEGA, employing the Maximum Likelihood (ML) method with the Tamura Nei nucleotide substitution model and a gamma distribution of rate heterogeneity among sites (Γ). For individual genes N, P, M, G and L, phylogenetic trees were obtained from deduced amino acid sequences using the ML method with the WAG + Γ amino acid substitution model [25]. The reliability of the different phylogenetic groupings was evaluated by considering 1000 bootstrap replications. The percent nucleotide and amino acid identities between the sequences were calculated using p-distances. The alignment of the N, P, M, G and L amino acid sequences of the CHPV isolates and other rhabdoviruses was done using the Profile Alignment Mode of ClustalX v.2.0.11 [26]. Profile alignment was done where the alignment of CHPV and other vesiculoviruses along with BEFV and SMRV was used as profile 1 and the lyssavirus alignment was taken as profile 2. Though all viruses as in the phylogenetic tree (Fig. 1) were used for the alignments, only representative viruses are included in all the alignment figures.

### Functional Motif Search And Tertiary Structure Modeling

The amino acid sequences of the N, P, M, G and L proteins were scanned for biologically important motifs using the Scanprosite tool of ExPaSy (http://expasy.org/tools/). Homology modeling of the N, M and G proteins was done with Modeler [27] in the Discovery Studio (DS) interface (Accelyrs Inc, USA). Models were energy minimized in DS employing the steepest descent followed by the conjugate gradient algorithms. The stereochemical quality of the energy minimized models was evaluated in PROCHECK [28]. The quality of the models was also evaluated using PROSA [29] (https://prosa.services.came.sbg.ac.at/prosa.php) and also using the neural network algorithm implemented in the PROQ server [30] (http://www.sbc.su.se/~bjornw/ProQ/ProQ.cgi). In PROSA, the quality scores of a protein are displayed in the context of all known protein structures while the PROQ predictor is based on a number of structural features and the protein quality is quantified by the location in the plane formed by the two indexes LGscore (i.e., the -log of a p-value) and MaxSub (ranging 0 – 1). Depending on the specific values of these indexes, the model can be qualified as: correct if LGscore>1.5 and MaxSub>0.1, as good if LGscore>3 and MaxSub>0.5, and as very good if LGscore>5 and MaxSub>0.8).

The crystal structure of VSV nucleoprotein in complex with RNA (PDB ID: 2GIC), which had 50.5% sequence identity and 71.6% sequence similarity with the N query sequence (GenBank Accession No: AY614724), served as the template for the CHPV ‘N’ protein model. The Ramachandran plot of the model (residues: 2 to 422) showed 98.7% residues in the allowed region, 0.8% in the generously allowed and 0.5% residues in the disallowed region. Further, validation of the minimized N protein model with ProSA indicated that the overall model quality was excellent (Z score of −8.05 in comparison with −8.34 of the template). The model was evaluated as “correct” according to the LG score (4.542) and MaxSub index (0.157).

The crystal structure of VSV ‘M’ protein (PDB ID: 1LG7) which shared 40.4% sequence similarity and 20.9% sequence identity with the M query sequence (GenBank Accession No: AF128868) was taken as the template to build the CHPV M protein homology model. The model (res: 61 to 229) had 98.7%, 0.7% and 0.7% residues in the allowed regions, additionally allowed regions and disallowed regions of the Ramachandran plot respectively. Validation of the predicted model with ProSA indicated that the overall quality of the minimized M protein model was very good (Z score of −6.36 in comparison with −5.24 of the template). The model was also evaluated as “correct” according to the LG score (4.51) and MaxSub index (0.27).

The G protein was modeled using 2J6J.PDB, the crystal structure of the prefusion form of VSV as the template, which shared 49.2% sequence similarity and 31.6% sequence identity with the G query sequence (GenBank Accession No: AY614717). The disulphide bridges for the model were specified according to the cystein linkages deduced by Walker and Kongsuwan for the structural model of rhabdovirus glycoproteins [31]. The Ramachandran plot of the model (residues: 22 to 526) showed 97.9% of the residues to be in allowed region, 1.2% of the residues in the generously allowed region and only 0.1% in the disallowed region. Further, ProSA indicated that the overall quality of the minimized G protein model was good (Z score of −4.32 in comparison with −7.47 of the template). The model was evaluated as “correct” according to the LG score (4.24) and MaxSub index (0.075). Solvent accessibility analysis of the G model was obtained by DSSP [32]. The model was submitted to the Conformational Epitope Prediction server [33] for the prediction of Antigenic Determinants (AD) and Conformational Epitopes (CE).

## Results

The whole genome lengths of the CHPV isolates, CIN0327 (AP, 2003), CIN0451 (Gujarat, 2004), CIN0755 (AP, 2007) and CIN0728 (Maharashtra, 2007) were 11,124, 11,124, 11,087 and 11,098 nucleotides respectively. The phylogeny of the whole genomes of the CHPV isolates and other rhabdoviruses (Fig. 1) showed CHPV to be closest to ISFV with 100% bootstrap support and mean nucleotide identity of 62%, followed by the other vesiculoviruses, (identity ranging from 51.2–55.4%), the SMRV (46.8%), BEFV (43.7%) and finally the lyssaviruses (41 – 42%). All the CHPV isolates including the prototype CIN6514 formed a monophyletic cluster with a 100% bootstrap value. CIN0327 and CIN0451 clustered together with ∼100% bootstrap support and so also CIN0728 and CIN0755. CIN0327 was found to be closest to the prototype strain CIN6514 with 96.46 percent nucleotide identity (PNI) followed by CIN0451 (96.36), CIN0728 (96.31) and CIN0755 (96.29).

Phylogenetic analyses of the proteins coded by the individual genes (Fig. S1) indicated similar topology as in the whole genomes. Except for the P protein, the clustering of the selected vesiculoviruses was supported by greater than 60% bootstrap support. The mean divergence among the rhabdoviruses selected varied from 48.7% for the L protein to 74.8% for the P protein, while the divergence among the vesiculoviruses varied from 29.7% to 57.3% for the L protein and P protein respectively. Fig. S2 further represents the comparison of the pairwise PNI and percent amino acid identity (PAI) among the CHPV isolates for the N, P, M, G and L genes/proteins.

The CHPV isolates of 2003–2007 exhibited several amino acid substitutions when compared with the prototype strain, CIN6514 (Table 1). In addition to the four whole genome isolates, the available partial G, N and P genes [22] of seven other isolates from the AP, 2003 outbreak (CIN0309R, CIN0318R, CIN0327R, CIN0327M, CIN0331M, CIN0360R, CIN0360V (GenBank accession nos. AY614718-AY614723 and AY614725-AY614731) were also included in the comparison. The importance and possible role of the mutations observed with respect to the prototype strain as well as with other rhabdoviruses are described here.

**Table 1:** Amino acid substitutions observed in the CHPV isolates with respect to the prototype strain CIN6514.

Protein	Polypeptide position	CIN 6514	CIN 0318R	CIN 0309R	CIN 0360V	CIN 0360R	CIN 0331M	CIN 0327M	CIN 0327M	CIN 0327	CIN 0451	CIN 0728	CIN 0755
**G**	16	I	.	.	V	V	.	.	.	.	.	.	.
	17	T	.	.	.	.	.	.	.	.	A	.	.
	19	L	S	S	S	S	S	S	S	S	S	S	S
	22	Y	S	S	S	S	S	S	S	S	S	S	S
	30	N	.	.	S	S	.	.	.	.	.	.	.
	40	R	K	K	K	K	K	.	.	K	K	K	K
	213	D	.	V	.	.	.	.	.	.	.	.	.
	218	I	.	.	V	V	.	.	.	.	.	.	.
	219	T	A	A	A	A	A	A	A	A	A	A	A
	222	G	A	A	A	A	A	A	A	A	A	A	A
	264	R	K	K	K	K	K	K	K	K	K	K	K
	269	H	P	P	P	P	P	P	P	P	P	P	P
	367	P	M	M	.	.	M	M	M	M	M	M	M
	424	L	.	.	.	.	.	V	V	V	.	.	.
	502	R	.	.	K	K	.	.	.	.	.	.	.
	503	K	.	.	.	.	.	.	.	.	.	R	.
**N**	37	K	R	R	R	R	R	R	R	R	R	R	R
	140	F	.	.	.	.	.	.	.	.	L	.	.
	163	A	.	T	.	.	.	.	.	.	.	.	.
	364	E	D	D	D	D		D	D	D	D	D	D
	413	V	.	.	I	I	.	.	.	.	.	.	.
**P**	64	E	D	D	D	D	D	D	D	D	D	D	D
	103	Q	.	R	.	.	.	.	.	.	.	.	.
	112	G	.	E	.	.	E	E	E	E	.	E	E
	180	I	.	.	V	V	.	.	.	.	.	.	.
	214	A	V	V	V	.	V	V	.	.	V	V	V
	221	M	.	.	.	.	.	.	.	.	I	.	.
	258	N	.	.	.	.	.	T	.	.	.	.	.
	270	I	V	V	.	.	V	.	.	.	V	V	V
													
**M**	97	D	----	----	----	----	----	----	----	.	.	N	.
**L**	52	D	----	----	----	----	----	----	----	.	N	.	.
	108	D	----	----	----	----	----	----	----	V	.	.	.
	115	E	----	----	----	----	----	----	----	.	.	D	.
	136	I	----	----	----	----	----	----	----	.	.	V	V
	139	V	----	----	----	----	----	----	----	I	.	.	.
	209	V	----	----	----	----	----	----	----	.	.	I	.
	298	I	----	----	----	----	----	----	----	.	V	.	.
	317	A	----	----	----	----	----	----	----	.	.	.	T
	320	P	----	----	----	----	----	----	----	.	.	.	Q
	408	T	----	----	----	----	----	----	----	.	.	A	A
	409	N	----	----	----	----	----	----	----	S	S	.	.
	586	E	----	----	----	----	----	----	----	D	D	.	.
	649	K	----	----	----	----	----	----	----	R	.	.	.
	652	I	----	----	----	----	----	----	----	.	.	.	L
	924	K	----	----	----	----	----	----	----	.	.	R	R
	1070	G	----	----	----	----	----	----	----	S	S	.	.
	1288	T	----	----	----	----	----	----	----	A	.	.	.
	1324	N	----	----	----	----	----	----	----	.	.	S	S
	1550	S	----	----	----	----	----	----	----	N	.	.	.
	1731	W	----	----	----	----	----	----	----	.	.	R	.
	1769	H	----	----	----	----	----	----	----	N	N	N	N
	1832	K	----	----	----	----	----	----	----	R	.	.	.
	1852	L	----	----	----	----	----	----	----	F	F	F	F
	1940	S	----	----	----	----	----	----	----	.	.	L	.
	1992	H	----	----	----	----	----	----	----	R	R	.	R
	2045	G	----	----	----	----	----	----	----	.	.	.	R
	2050	N	----	----	----	----	----	----	----	S	S	S	S

A ‘dot’ indicates a match with the amino acid in CIN6514 and a dash indicates non-availability of sequence data. Whole genome isolates of this study are underlined.

doi:10.1371/journal.pone.0030315.t001

### Nucleocapsid Protein

**Figure 2:**
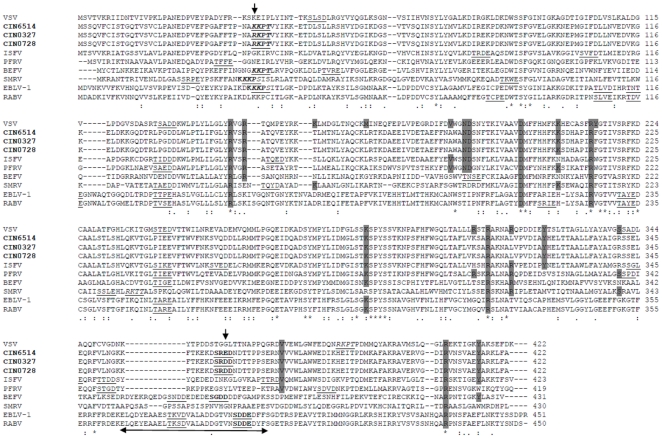
Profile alignment of N protein of representative CHPV isolates (shown in bold) with other representative rhabdoviruses. Residues known to be critical for VSV N protein function are highlighted in grey to indicate conservation. Putative CK II phosphorylation sites are underlined while putative CAMP phosphorylation sites are italicized and underlined. The two-headed arrow delineates the T cell epitope of lyssaviruses. Mutations in CHPV isolates are indicated by downward arrows.

A putative casein-type phosphorylation site [ST]X(2)[DE] at Ser389 [34] that is conserved in all lyssaviruses (as SXXE) and also shown to be crucial for viral RNA transcription and replication [35] was noted in CHPV (as SRDD) at equivalent position 362 but not in other vesiculoviruses (Fig. 2).

**Figure 3:**
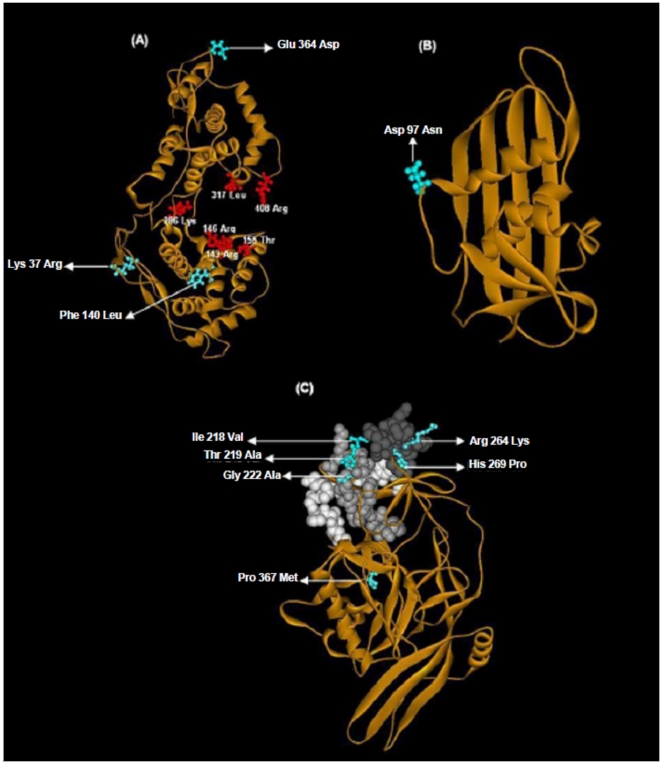
Homology models of CHPV proteins and mapping of significant functional residues. All observed CHPV mutations (residue numbering as per the location in the sequence) in cyan. (A) Nucleoprotein: RNA binding residues shown in orange, residues part of RNA binding groove in blue and loop Ser340-Val375 in yellow. (B) Matrix (C) Glycoprotein: BEFV antigenic site G3 shown in shades of grey.

CHPV N shared 50.5% identity with the N protein of VSV, its closest neighbour, the crystal structure of which was determined recently [36]. A single chain of the N-RNA crystal structure of VSV (PDB ID: 2GIC) was taken as the template to model the nucleoprotein of CHPV (Fig. 3A) to gain insights into the N-N interactions which stabilize the nucleocapsid assembly [15]. The CHPV ‘N’ model comprised of 21 helices and 8 strands and showed an overall RMSD of 0.05Å with respect to the template. The Ser340-Val375 loop in the VSV structure, which extends to interact with the adjacent molecule in the N protein ring, was maintained as a loop in the model (Arg340-Val375) (Fig. 2) with a RMSD of 0.95 Å with respect to the template. This thirty five-residue stretch was conserved in all the CHPV isolates.

Residue Arg143 from the VSV N terminal lobe, involved in binding to the phosphates group of RNA was conserved in the CHPV isolates as also in all the rhabdoviruses included in this study (Fig. 2). Arg146 also involved in RNA binding was conserved in all vesiculoviruses and BEFV, with a homologous substitution Lys146 noted in the lyssaviruses. Another interacting residue from the same lobe Lys155 in VSV was changed to ‘Thr’, a non conservative change in all the CHPV isolates. RNA binding residues from the C-terminal lobe of VSV nucleoprotein, Lys286 and Arg408 were strictly maintained in all the rhabdoviruses studied whereas another interacting residue Arg317 was replaced by Leu317 in all the CHPV isolates in the same lobe. Two additional residues, Arg214 and Arg312 placed in the RNA binding cavity but not making contact with the RNA in VSV were conserved in all the rhabdoviruses. Asp199 which is bonded to Arg214 by a salt bridge was also conserved. Tyr215, which stacks against nucleotide 1 of RNA, was conservatively substituted by Phe215 in all the CHPV isolates. Tyr324 and Tyr415 from the C-terminal lobes that interact with the hydrophobic portion of the side chain of the Arg309 were conserved in CHPV and ISFV though the Arg309 of VSV was substituted by Lys309, a homologous residue in CHPV. The Glu419 which neutralizes the positive charge of Arg309 in VSV was replaced by a ‘Lys’ in CHPV at the same position. Except Met166, residues Lys207, Asn187 and Asp188 responsible for maintaining the hydrophobic interaction between the N-terminal lobes were maintained in CHPV and most vesiculoviruses. Val184 also involved in the same interaction was substituted by a similar residue Ala184 in CHPV.

When compared with CIN6514 two amino acid substitutions, namely, Lys37Arg and Glu364Asp (except in CIN0331M) were observed in the 2003–2007 isolates (Table 1). The Lys37Arg mutation appeared within the N-terminal region containing the first 47 amino acid residues which have been shown to be critical for self assembly of N [16]. The same mutation was located in a putative CAMP phosphorylation motif [RK](2)X[ST] at residue position 37 (with the phosphorylation site at 40Thr) in the CHPV isolates. The mutation however being in the first position of the motif, the phosphorylation site was maintained. This phosphorylation site was conserved at the equivalent position in several rhabdoviruses including BEFV, European bat lyssavirus-1 (EBLV-1), European bat lyssavirus-2 (EBLV-2), Irkut virus (IRKV), Australian bat lyssavirus (ABLV) and Aravan virus (ARAV) but not VSV and ISFV (Fig. 2). The Glu364Asp substitution appeared within the C-terminal region shown to be important for N-RNA interaction [16] and also the stretch Arg340-Val375, important for N-N interactions in VSV. It was further found to map onto the amino acid stretch 373–395 (region 357–366 in CHPV) of the lyssaviruses, delineated as an important T-cell epitope in humans and mice [34]. This stretch was also predicted as a T-cell epitope in CHPV (data not shown) by predictive methods, SYFPEITHI [37] and HLA_Bind [38]. Though the mutation also appears in the putative Casein phosphorylation site Ser362, the phosphorylation motif is maintained.

### Phosphoprotein

**Figure 4:**
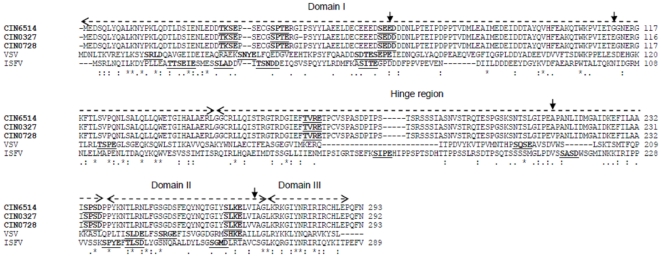
Alignment of P protein of representative CHPV isolates (indicated in bold) with closely related vesiculoviruses VSV and ISFV. All putative phosphorylation sites are underlined. The different domains defined for the P protein of VSV are indicated by two-headed dashed overhead arrows. The mutations within the CHPV isolates are indicated by downward arrows.

In general three domains and a hypervariable hinge region have been described in the P protein of VSV [39]. Domain 1 is responsible for the association of P with L and needs to be phosphorylated in several sites for optimal transcription activity [40]. A Casein Kinase II (CK II) phosphorylation motif, [ST]X(2)[DE], was found in the prototype CHPV isolate where Ser62 is the phosphorylation site (Fig. 4) which is specifically modified by CKII [41]. The P protein alignment of the CHPV isolates with other rhabdoviruses (sup link: Fig. S3) indicated that phosphorylation motifs were found at more or less equivalent positions in VSV (Ser60, Thr62 and Ser64), ISFV (Ser56) and SVCV (Ser64). Several residues are conserved between CHPV, VSV and also ISFV in domains II and III (Fig. 4). On the other hand, the region in CHPV corresponding to the VSV hinge region appears to be least conserved. The short lysine-rich motif (FSKKYKF) of lyssaviruses critical for ribonucleoprotein (RNP) binding [42] was not noted in CHPV and the other rhabdoviruses included in this study (Fig. S3). The dynein light chain 8 (LC8) binding motif [KR]XTQT of the lyssaviruses [43],[44] that had earlier been suggested as a molecular factor that links viral RNP to the host cell transport system in RABV [45] was also not found to be maintained in the vesiculoviruses and other rhabdoviruses studied here. More recent studies [46] have however proposed that LC8 does not play a role in the retrograde axonal transport of RABV, but may have a role in promoting efficient viral transcription.

An overlapping ORF (nucleotide position 1445–1687/length 243 nt) in the +2 reading frame was predicted within the P gene (nucleotide position 1363 – 2244/length 882 nt) of CHPV using the tool ‘ORF Finder’ at NCBI (http://www.ncbi.nlm.nih.gov/projects/gorf/).

Among eight mutations observed in the 2003–2007 isolates, a Glu64Asp mutation was observed in all the isolates (Table 1). It was found to be present in the third position of the CK II phosphorylation site. The sequence motif in these isolates was SEDD while that in CIN6514 was SEED and thus the phosphorylation motif is maintained. The significance of the mutations Gly112Glu, Ala214Val and Ile270Val, in several isolates could not be ascertained.

### Matrix Protein

**Figure 5:**
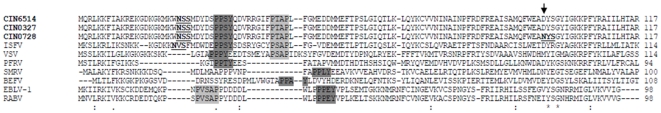
Profile alignment of M protein (res: 1 – 117) of representative CHPV isolates (indicated in bold) with other representative rhabdoviruses. The conserved PPxY and Px[TS]AP motifs are highlighted in grey. Putative glycosylation sites are underlined. The mutations within the CHPV isolates are indicated by downward arrows.

The N terminus of the matrix protein of all the CHPV isolates contained a highly conserved PPSY sequence (position: 30) as was also noted in ISFV. This was similar to the PPPY motif in VSV and PPxY motif of other rhabdoviruses except Khujand virus (KHUV) (Fig. 5), implicated to be involved in the late stage in virus budding [47]. Another core motif P[ST]AP reported to bind components of vascular protein sorting pathway, such as tumor susceptibility gene 101 (Tsg 101) [48], was observed to be strictly conserved PTAP in all the CHPV isolates at position 42 as also in other vesiculoviruses (PSAP) except pike fry rhabdovirus (PFRV) and spring viraemia of carp virus (SVCV). The motif was seen to be Px[TS]AP in some of the lyssaviruses at position 21. The composition of amino acids surrounding the late budding domain (L-domain) core motifs, which are critical for efficient L-domain activity and for interactions with host proteins in the context of VSV infection [49] though found to be conserved in all the CHPV isolates differs from the amino acid composition in VSV and is more similar to that of ISFV.

At the amino acid level, M protein was found to be totally conserved in all the isolates but CIN0728 (Fig. 5). This isolate possessed an Asp97Asn substitution, which resulted in the gain of a putative N-glycosylation site. A homology based model of the M protein (Fig. 3B) based on the crystal structure of the VSV M protein [50] showed that the Asp97Asn mutation was located in a loop region.

### Glycoprotein

**Figure 6:**
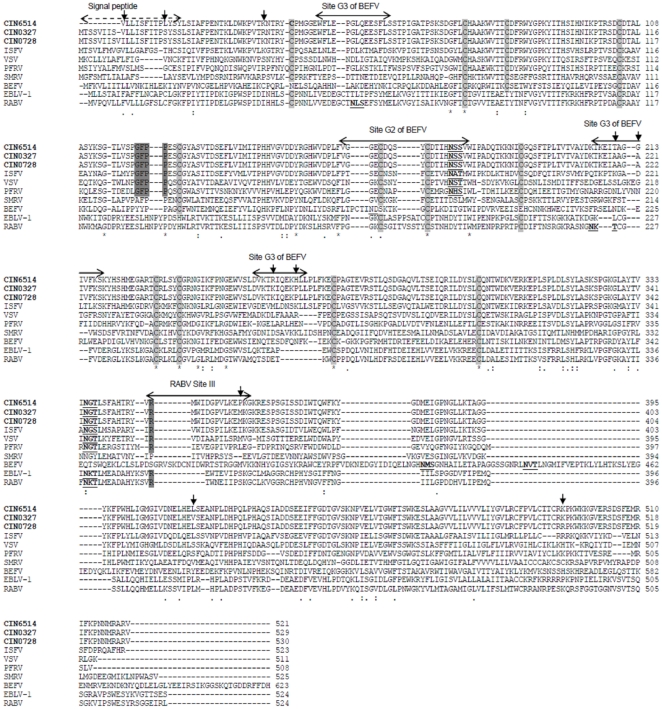
Alignment of G protein of representative CHPV whole genome isolates (in bold) with other representative rhabdoviruses. BEFV antigenic sites and RABV antigenic sites are indicated by arrows above the sequence. Functionally important motifs are highlighted in grey and putative glycosylation sites are underlined. The signal peptide is indicated by dashed arrow. Highly conserved cysteine residues are highlighted in faint grey. Mutations within CHPV isolates are indicated by downward arrows.

The GFPP motif involved in fusion [51] was conserved in all the CHPV isolates at position 129 as in all the other vesiculoviruses (Fig. 6). The residue corresponding to Arg333 in RABV, within the rabies antigenic site III and reported to be involved in neuropathogenicity [52] was found to be a conserved Arg356 in all the CHPV isolates as in all vesiculoviruses and many of the lyssaviruses considered in this study.

The G protein of all CHPV isolates possessed two putative glycosylation sites at positions 175 and 335. Of these, the first site that appears to be associated with antigenic site G2 of BEFV [53] was found to be present in all other vesiculoviruses while the second one was present in all rhabdoviruses except SMRV and BEFV.

Among all the major viral proteins, the G protein was the least conserved with PAI varying from 98.47–99.81 (Fig. S2). With respect to the prototype isolate, eleven amino acid mutations, Thr17Ala (only in CIN0451), Leu19Ser, Tyr22Ser, Lys40Arg (in CIN0327), Thr219Ala, Gly222Ala, Arg264Lys, His269Pro, Pro367Met, Leu424Val, Lys503Arg (only in CIN0728) were noted in the 2003–2007 whole genome isolates (Table 1) and six of these substitutions (Leu19Ser, Tyr22Ser, Thr219Ala, Gly222Ala, Arg264Lys, His269Pro) were recorded in the other 2003 partially sequenced isolates also. Of these six mutations Thr219Ala, Gly222Ala, Arg264Lys, and His269Pro could be mapped (Fig. 6) onto antigenic site G3 of BEFV [53],[54], and equivalent site G of RABV [55]. The mutations, Leu19Ser and Tyr22Ser were not part of any antigenic site. A substitution Pro367Met could be mapped within the RABV antigenic site III [52],[55],[56]. A substitution Ile218Val also in antigenic site G3 was observed in two of the 2003 partially sequenced isolates.

A homology model of the ‘G’ ectodomain (Fig. 3C) was built to study the spatial distribution of the antigenic stretches bearing the critical mutations, on the three dimensional structure of the protein. The three discontinuous amino acid stretches (res: 53–63, 215–229, 260–273) of antigenic site G3 were observed to be spatially close in the G protein model. Critical mutations Thr219Ala, Gly222Ala, Arg264Lys, and His269Pro were found to be surface exposed. Three of these amino acid residue stretches also featured in the predicted antigenic determinants (AD Nos. 9 and 11 in Table S2A) comprising two conformational epitopes [213–222, 263–267] (CE No 8) and [263–267, 270–284] (CE No 10) in Table S2B.

### Large Protein

Invariant residues in the L protein are embedded into blocks of conserved stretches separated by variable regions [57]–[59]. Six such blocks have been defined for Rhabdoviruses and paramyxoviruses [60],[61]. The alignment of the CHPV with other rhabdoviruses was done (Fig. S4) to note the degree of conservation of these blocks in the CHPV isolates. The equivalent Block I in CHPV contained hydrophobic residues particularly in the first two conservative stretches (res: 219–230 and res: 269–289). The third conservative stretch (res: 340–354) in the block I of CHPV showed an invariant GHP motif (res: 349–351) as in all the rhabdoviruses studied here. Blocks II and III consist of the major functional domains. The pre-A motif which has been shown to be involved in the positioning and binding of the RNA template [62] as well as the KERE motif [61] were found to be well conserved among all the rhabdoviruses included in this study. The four conserved regions in negative-stranded RNA virus L proteins (A–D) in block III [61] were conserved in the CHPV isolates and also well conserved among the other rhabdoviruses. The GG[IL]EG (668–672) motif and the invariant pentapeptide QGDNQ (702–706) with precise spacing between them, in the block III as noted earlier in measles virus [63] were strictly conserved in all the CHPV isolates as in the other rhabdoviruses. Block V showed numerous cysteine and histidine invariant residues. Block VI showed the GXGXG motif as GDGSG sequence in the CHPV isolates (res: 1659–1663) as also noted in other rhabdoviruses, preceded by a lysine 19 residues upstream, which could play the role of polyadenylation or protein kinase activity [60].

The HR motif [64] at position 1217 which has been shown to be necessary for the PRNT ase activity of the L protein, at the step of enzyme-pRNA intermediate formation [61], was found to be conserved in all the CHPV isolates as in other rhabdoviruses and the equally important R in the vicinity of the HR motif at position 1211 was also conserved in the isolates. The same was also conserved in the other rhabdoviruses studied here. An experimental CTL epitope IRRA noted in protozoa [65] was conserved in all the CHPV isolates at position 1166 and was also conservatively maintained in other vesiculoviruses (IKRA) and rhabdoviruses (VKRA).

The L protein of the 2003–2007 isolates, exhibited the maximum number of mutations with respect to the prototype isolate (Table 1). A Thr→Ala mutation was observed in CIN0327 at position 1288, immediately adjacent to amino acid residues that correspond to a strictly conserved stretch (res: 1284–1287) [60]. A Ser1550Asn mutation was observed only in CIN0327. The Thr1288Ala substitution can lead to the loss of a potential protein kinase C phosphorylation site while a Ser1940Leu substitution in CIN0728 can result in a loss of a putative CAMP phosphorylation site at position 1937. Considering the potential problem of overpredicition of phosphorylation sites, we looked into the prediction in other vesiculoviruses as well. None of the related viruses had any phosphorylation site predicted at equivalent positions and hence the significance of the same in CHPV may be limited.

### Genomic Termini

The leader sequence nucleotides were highly conserved among all the CHPV isolates. With respect to the prototype isolate, two nucleotide changes, A→T at position 19 was observed in all the isolates and A→C was observed at position 37 in CIN0451. The trailer terminus showed two nucleotide substitutions, T→C in CIN0327 and A→T in CIN0755 at positions 1 and 10 respectively, from the trailer start (Fig. S5).

### Non-Coding Regions And Gene Junctions

The non-coding N-P, P-M and M-G gene junctions contained the conserved sequence, 3′AUAC (U)7 NNUUGUC 5′ in all the CHPV isolates. The gene start sequence which immediately follows the intergenic dinucleotide (NN) was conserved in all the isolates for every gene junction (Table 2).

**Table 2:** Intergenic dinucleotide for all the gene junctions (3′ → 5′ direction) in CHPV whole genome isolates.

Gene Junction	CIN6514	CIN0327	CIN0451	CIN0728	CIN0755
N–P	GA	GA	GA	GA	GA
P–M	AA	AA	AA	AG	AG
M–G	GA	AA	AA	AA	AA
G–L	GA	GA	GA	GA	GA

doi:10.1371/journal.pone.0030315.t002

With respect to the prototype isolate, a substitution G→A was observed at the first position of the M–G junction intergenic dinucleotide of all the 2003–2007 isolates and A→G substitution in the P-M junction dinucleotide was noted in CIN0728 and CIN0755 in the second position. A block of 18 nucleotide insertion in the G–L junction of CHPV, between the non-transcribed dinucleotide (CU) and the consensus start sequence, as reported earlier [66],[67] showed changes at only two nucleotide positions in CIN0451, CIN0728 and CIN0755.

## Discussion

The phylogenetic analyses of the whole genomes and the proteins coded by the individual genes indicated that the CHPV isolates clustered with the ISFV. The percent nucleotide identities amongst the five CHPV isolates of the study revealed that the virus was relatively stable over the period of 42 years (1965–2007). The percent nucleotide divergence of the CHPV whole genomes varied from 3.54–3.71, with respect to the prototype. The clustering pattern among the CHPV isolates observed in the phylogenetic tree brought out a role of temporal factors rather than geographic factors in the evolution of CHPV. This pattern was reflected in the amino acid substitutions as well (Table 1) with CIN0327 and CIN0451 sharing common mutations just like CIN0755 and CIN0728, many of them in the L protein. Several functional motifs characteristic of CHPV were conserved within all the isolates. In addition, the isolates also maintained functional motifs observed in other rhabdoviruses.

Comparison of rhabdovirus and paramyxovirus N proteins had revealed a region with significant identity at the center of the polypeptide [67] implicated for N-N association [15]. Crystallographic data on VSV-N showed that this central region forms constituents of both N- and C- terminal lobe, which come together to form a cavity that accommodates the RNA [36]. The residues in this central region of the N protein in the CHPV isolates were highly conserved as has also been observed in several other members of the *Rhabdoviridae* family. The overall architecture of the binding cavity in the N protein was thus maintained in CHPV.

The Lys37Arg mutation in the N protein of the 2003–2007 isolates, was within the first 47 amino acids that was determined to have an important role in the oligomerization of N protein in CHPV [16]. The implication of this homologous substitution might not be significant in terms of monomeric N-N interaction. Also, though the mutation occurred within a phosphorylation motif, the putative phosphorylation site 40Thr was conserved. Further, the homologous substitution Glu364Asp in the CHPV isolates was located within the C-terminal region (320–422 aa) and might not affect RNA recognition [16]. However, as it mapped on to a probable T-cell epitope, it needs to be experimentally verified for pathogenicity. The mutation also corresponds to the last 60 amino acids of the C-terminal end of VSV N protein where sequences known for interaction with the phosphoprotein and encapsidation are believed to be located [68]. Further it mapped on to a putative CKII phosphorylation site that was noted only in CHPV among the vesiculoviruses studied here and the lyssaviruses. Therefore, the implication of Glu→Asp mutation requires careful examination. Interestingly, this mutation also positioned within the thirty five residue loop in the N model corresponding to the VSV loop responsible for the interaction with the adjacent N protein ring. Overall the site may be a feature distinguishing CHPV and VSV.

Among all proteins, the P protein was found to be the least conserved for the selected rhabdoviruses as well as for the selected vesiculoviruses. This can explain the lower bootstrap supports observed for the vesiculovirus cluster, in case of the P protein phylogeny. Further, though the length of the P protein of CHPV and VSV differed significantly, yet domains I, II and III aligned fairly well with gaps noted mainly in the hinge region. Conserved residues in domains II and III are important for P protein function in mediating the binding of P protein to N-RNA template while the hypervariable hinge region of the P protein plays an important role in viral RNA synthesis and in the assembly of infectious VSV [39], hence the role of the same in CHPV needs further investigation. An additional ORF could be predicted within the P gene of the CHPV isolates as was previously identified or predicted in the P genes of VSV [69], other vesiculoviruses [23],[70] and SMRV [71]. However, the expression of the same in CHPV remains to be verified experimentally.

Further, in the P protein, a Glu64Asp substitution was maintained consistently in the 2003–2007 isolates which was found to be located in the known CKII phosphorylation site. Additional acidic residues in the CK II phosphorylation motif at positions +1, +2, +4, and +5 from the start of the phosphorylation site were observed to increase the rate of phosphorylation and Asp is preferred to Glu as the provider of acidic determinants [72]. Therefore the Glu→Asp substitution could have an impact on the rate of phosphorylation and hence altered biology of the P protein as CKII mediated phosphorylation is indispensable for CHPV P to act as a transcriptional activator [73]. Also, this phosphorylation induces a major structural change in the N terminal domain (res: 49–69) of the P protein [18], which is clearly known to be important in L protein interaction for *vesiculoviruses* [40]. Implication of the Glu64Asp mutation merits further experimentation to understand its role in pathogenicity of the 2003-07 CHPV isolates.

Significant motifs including those involved in virus budding were identified in the M protein of all CHPV isolates as in most of the other rhabdoviruses. However, the differences in the L-domain flanking sequences specifically with respect to VSV may have implication for interaction with host proteins. Notably both CHPV and ISFV [74] infect humans unlike VSV. The only mutation, Asp→Asn at position 97 in the M protein which was observed in CIN0728 resulted in the gain of a putative glycosylation site. The significance of this mutation in terms of pathogenesis also requires to be explored.

The fusion motif in the G protein was conserved in all the CHPV isolates as also in other vesiculoviruses. The homology model of the G protein ectodomain of CHPV was also built to study the antigenic fine structure. The model revealed that the sequence stretches of the BEFV antigenic site G3 which are also known neutralization epitopes of VSV [53],[75], clustered in the three-dimensional structure of the protein, pointing to the probable implication of the site in presenting an antigenic face in CHPV. The same also matched with one of the predicted conformational epitopes (CE), implying that it is likely to be a major antigenic site (Table S3B). The critical mutations in the 2003–2007 isolates could be mapped onto this CE and were exposed so as to enable interaction with cellular receptors and antibodies, suggesting that these residues may be under immune selection pressure. Interestingly, amongst the critical mutations, the Leu19Ser, Tyr22Ser and Thr219Ala, His269Pro are non conservative changes. The last two substitutions that are located in the antigenic sites, involve a change from polar residues to non polar and hydrophobic residues. These changes may affect antibody binding. However other studies in the author's laboratory [76] have shown that antibodies induced by a recombinant G protein based vaccine could efficiently neutralize the different CHPV viruses with neutralizing titers ranging from 70 to 120 (*pers. comm*.) suggesting that these mutations may not significantly alter immunoreactivity.

The invariant amino acids were not distributed randomly in the L proteins, but were clustered into the six blocks defined previously [60], compatible with the concept that the L protein needs to have concatenated functional domains for performing many enzymatic functions. [59]. The conservation of the GHP motif in Block I implies the conservation of architecture of this functional domain in CHPV. Presence of charged conserved stretches throughout block III around the pentapeptide QGDNQ again points to an important functional role for the entire block III. The pentapeptide domain is proposed to be the active site for template recognition and/or phosphodiester bond formation in the L proteins [60] and has been extensively studied by utilizing deletions and mutations. Any change made to the motif resulted in complete loss in polymerase activity in RABV [77] and VSV [78]. The sole change Ser1550Asn in CIN0327, within a defined conserved block, requires this mutation to be looked into.

The non-coding genomic termini of *vesiculoviruses* have been shown to be maintained with blocks of strong conservation [23]. The first 21 nucleotides of the (+) leader RNA is specifically recognized by the nucleocapsid protein monomer which is followed by nucleocapsid assembly. Subsequent N-N association during elongation phase results in subtle conformational changes to allow for compromised binding specificity so that the polymerized N can bind to heterogeneous sequences [79]. Therefore the observed A→T substitution in all the 2003–2007 CHPV isolates at position 19 from the leader terminus might not have significant effect on the binding specificity of N.

The gene junctions of CHPV were very similar to those of ISFV and VSV, as had been observed earlier [23],[80]. The intergenic conserved sequence 3′AUAC U(7)NNUUGUC5′ in the CHPV gene junctions had also been reported by Marriott [23]. The stretch 3′AUAC U(7) 5′ was strictly conserved in every gene junction among all the CHPV isolates as it is critical for both termination and polyadenylation of mRNA and mutations are rarely observed at these sites as has been shown by *in vitro* studies [81].

The U(7) polyadenylation signal was followed by two non transcribed nucleotides 3′-NN-5′ (-G/CA-) which act as an essential element for efficient transcription termination [81], signal for initiation, capping and methylation of downstream mRNA. The intergenic dinucleotide of the M-G gene junction in the 2003–2007 isolates differed from the prototype strain, at the first position. Substitutions at the first position have been found to have an enhancing impact on the amount of readthrough transcripts as well as the transcription of the upstream gene in VSV [82]. It would therefore be interesting to quantify the M–G junction readthrough as well as gene transcripts in CHPV. The other change A→G, in the P–M junction intergenic dinucleotide which was noted in CIN0728 and CIN0755 appeared in the second position. Though single substitutions at the second position were found to result in only slightly increased levels of read-through transcripts [83], they are also found to play a small role [84]. It would be worth studying whether this trend observed in 2007 will be maintained over time.

Variations in the G–L intergenic region just following the dinucleotide (NN) were observed earlier in CHPV [23]. The same trend had been observed in other viruses as well [81],[82],[65], and the insertion sequence was hypothesized to be vestigial [65]. Therefore, the variations within the 18 nucleotide insertion in the G–L intergenic region amongst the 2003–2007 CHPV isolates observed in this study, possibly does not play any significant biological role.

Overall, this study presents the whole genome-based characterization of CHPV isolates from different encephalitis epidemics at different time points during 2003–2007 as well as comparison with the prototype 1965 isolate and the genomes of related rhabdoviruses. Several functional motifs, indicative of pathogenicity, were found to be conserved in all the CHPV isolates of this study. The specific regions and sites of differences with VSV may help in furthering our understanding of host specificity. Over the last four decades, the CHPV virus also showed significant mutations in the G, N, P, M proteins and the gene junctions. Our study can further form the basis for experimental verification by functional assays to understand the difference in pathogenicity between the 1965 virus and the more recent CHPV virus isolates and thus provide important insights into the virulence determinants of the CHPV.

## Supporting Information

Figure S1 **Protein-based phylogenetic trees of the genes of the CHPV whole genome isolates and other rhabdoviruses using the Maximum likelihood method as implemented in MEGA. v.5.05.** Bootstrap values greater than 60% are indicated at the appropriate nodes. Scale bar indicates number of nucleotide substitutions per site. (TIF)

Figure S2 **Pairwise percent nucleotide identity (PNI) and percent amino acid identity (PAI) among CHPV whole genome isolates.**
(TIF)

Figure S3 **Profile alignment of P protein of representative CHPV isolates (indicated in bold) with other representative rhabdoviruses.** All putative phosphorylation sites are underlined. The LC8 motif ([KR]XTQT) and the lysine rich motif (FSKKYKF) in lyssaviruses are highlighted in grey. The different domains defined for the P protein of VSV are indicated by arrows. The mutations within the CHPV isolates are indicated by downward arrows. (TIF)

Figure S4 **Profile alignment of the L protein of representative CHPV isolates (indicated in bold) with other representative rhabdoviruses.** Defined blocks of high conservation designated by roman numerals are shown in different colors. The conserved motifs, pre-A, A, B, C and D, are indicated by 2-headed arrowed lines. The nearly uninterrupted stretches of strictly or conservatively maintained amino acids (Poch et al., 1990) are indicated by red colored overhead lines. Functionally important motifs/residues are highlighted in grey. Mutations within CHPV isolates are indicated by downward arrows and associated potential phosphorylation sites are underlined. (PDF)

Figure S5 **Leader and trailer sequences in the CHPV whole genome isolates.**
(TIF)

Table S1 **Primers used for the amplification and sequencing of the CHPV whole genomes.**
(PDF)

Table S2 **Predicted antigenic determinants (A) and conformational epitopes (B) in the G protein of CHPV.**
(PDF)

## References

[1] Rodrigues FM, Patnakar MR, Banerjee K, Bhatt PN, Goverdhan MK (1972). Etiology of the 1965 epidemic of the febrile illness of Nagpur city, Maharashtra State, India.. Bull Wld Hlth Org.

[2] Bhatt PN, Rodrigues (1967). Chandipura: a new arbovirus isolated in India from patients with febrile illness.. IJMR.

[3] Rao BL, Basu A, Wairagkar NS, Gore MM, Arankalle VA (2004). A large outbreak of acute encephalitis with high fatality rate in children in Andhra Pradesh, India, in 2003 associated with Chandipura virus.. Lancet.

[4] Chadha MS, Arankalle VA, Jadi RS, Joshi MV, Thakare JP (2005). An outbreak of Chandipura virus encephalitis in the eastern districts of Gujarat state, India.. Am J Trop Med.

[5] Gurav YK, Tandale BV, Jadi RS, Gunjikar RS, Tikute SS (2010). Chandipura virus encephalitis outbreak among children in Nagpur division, Maharashtra, 2007.. Indian J Med Res.

[6] Tandale BV, Tikute SS, Arankalle VA, Sathe PS, Joshi MV (2007). Chandipura virus- a major cause of acute encephalitis in children in north Telangana, Andhra Pradesh, India.. J Med Virol.

[7] Basak S, Mondal A, Polley S, Mukhopadhyay S, Chattopadhyay D (2007). Reviewing Chandipura: A Vesiculovirus in Human Epidemics.. Biosci Rep.

[8] Kemp GE (1975). Viruses other than arenaviruses from West African wild mammals.. Bull World health Organ.

[9] Peiris JS, Dittus WP, Ratnayake CB (1993). Seroepidemiology of dengue and other arboviruses in a natural population of toque macaques (Macaca sinica) at Polonnaruwa, Sri Lanka.. J Med Primatol.

[10] Dhanda V, Rodrigues FM, Ghosh SN (1970). Isolation of Chandipura virus from sandflies in Aurangabad.. Indian J Med Res.

[11] Geevarghese G, Arankalle VA, Jadi R, Kanojia PC, Joshi MV (2005). Detection of chandipura virus from sand flies in the genus Sergentomyia (Diptera: Phlebotomidae) at Karimnagar District, Andhra Pradesh.. Indian J Med Entomol.

[12] Mullen G, Mullen GR, Durden L Medical and Veterinary Entomology (Elsevier, 2002) pg.

[13] Jadi RS, Sudeep AB, Kumar S, Arankalle VA, Mishra AC (2010). Chandipura virus growth kinetics in vertebrate cell lines, insect cell lines and embryonated eggs.. Indian J Med Res.

[14] Iverson LE, Rose JK (1981). Localized attenuation and discontinuous synthesis during vesicular stomatitis virus transcription.. Cell.

[15] Banerjee AK (1987). Transcription and replication of rhabdoviruses.. Microbiol Rev.

[16] Mondal A, Bhattacharya R, Ganguly T, Mukhopadhyay S, Basu A (2010). Elucidation of functional domains of Chandipura virus Nucleocapsid protein involved in oligomerization and RNA binding: implication in viral genome encapsidation.. Virology 10;.

[17] Chattopadhyay D, Chattopadhyay D (1994). Cloning of the chandipura virus phosphoprotein encoding gene and its expression in Escherichia coli.. Cell Mol Biol Res.

[18] Raha T, Chattopadhyay D, Roy S (1999). A phosphorylation-induced major structural change in the N terminal domain of the P protein of Chandipura virus.. Biochem.

[19] Raha T, Samal E, Majumdar A, Basak S, Chattopadhyay D (2000). N-terminal region of P protein of Chandipura virus is responsible for phosphorylation-mediated homodimerization.. Protein Eng.

[20] Kopecky SA, Lyles DS (2001). The Cell-Rounding Activity of the Vesicular Stomatitis Virus Matrix Protein Is due to the Induction of Cell Death.. J Virol.

[21] Le Blanc I, Luyet PP, Pons V, Ferguson C, Emans N (2005). Endosome-to-cytosol transport of viral nucleocapsids.. Nat Cell Biol.

[22] Arankalle VA, Shotri SP, Walimbe AM, Hanumaih, Pawar SD (2005). G, N and P Gene-based Analysis of Chandipura Viruses, India.. EID.

[23] Marriott AC (2005). Complete genome sequences of Chandipura and Isfahan vesiculoviruses.. Arch Virol.

[24] Kumar S, Tamura K, Nei M (2004). MEGA3: Integrated software for Molecular Evolutionary Genetics Analysis and sequence alignment.. Briefings in Bioinformatics.

[25] Whelan S, Goldman N (2001). A general empirical model of protein evolution derived from multiple protein families using a Maximum-Likelihood approach.. Mol Biol Evol.

[26] Thompson JD, Gibson TJ, Plewniak F, Jeanmougin F, Higgins DG (1997). The CLUSTAL_X windows interface: flexible strategies for multiple sequence alignment aided by quality analysis tools.. Nucleic Acids Res.

[27] Sali A, Blundell TL (1993). Comparative protein modelling by satisfaction of spatial restraints.. J Mol Biol.

[28] Laskowski RA, MacArthur, MW, Moss, DS, Thornton, JM (1993). PROCHECK - a program to check the stereochemical quality of protein structures.. J App Cryst.

[29] Wiederstein M, Sippl MJ (2007). ProSA-web: interactive web service for the recognition of errors in three-dimensional structures of proteins.. Nuc Acids Res July; 35 (Web Server issue).

[30] Wallner B, Elofsson A (2003). Can correct protein models be identified?. Protein Sci.

[31] Walker PJ, Konsuwan K (1999). Deduced structural model for animal rhabdovirus glycoproteins.. J Gen Virol.

[32] Kabsch W, Sander C (1983). Dictionary of protein secondary structure: Pattern recognition of hydrogen-bonded and geometrical features.. Biopolymers.

[33] Kulkarni-Kale, U, Bhosle S, Kolaskar AS (2005). CEP: a conformational epitope prediction server Nucleic Acids Res.

[34] Dietzschold B, Lafon M, Wang H, Otvos L, Jr Celis E (1987). Localization and immunological characterization of antigenic domains of the rabies virus internal N and NS proteins.. Virus Res.

[35] Yang J, Koprowski H, Dietzschold B, Fu ZF (1999). Phosphorylation of rabies virus nucleoprotein regulates viral RNA transcription and replication by modulating leader RNA encapsidation.. J Virol.

[36] Green TJ, Luo M (2006). Resolution improvement of X-ray diffraction data of crystals of a vesicular stomatitis virus nucleocapsid protein oligomer complexed with RNA.. Acta Crystallogr D Biol Crystallogr.

[37] Rammensee HG, Bachmann J, Emmerich NN, Bachor OA, Stevanovic S (1999). SYFPEITHI: database for MHC ligands and peptide motifs.. Immunogenetics.

[38] Parker KC, Bednarek MA, Coligan, JE (1994). Scheme for ranking potential HLA-A2 binding peptides based on independent binding of individual peptide side-chains.. J Immunol.

[39] Das SC, Pattnaik AK (2005). Role of the hypervariable hinge region of phosphoprotein P of Vesicular stomatitis virus in viral RNA synthesis and assembly of infectious virus particles.. J Virol.

[40] Pattnaik AK, Hwang L, Li T, Englund N, Mathur M (1997). Phosphorylation within the amino-terminal acidic domain I of the phosphoprotein of vesicular stomatitis virus is required for transcription but not for replication.. J Virol.

[41] Chattopadhyay D, Raha T, Chattopadhyay D (1997). Single serine phosphorylation within the acidic domain of Chandipura virus P protein regulates the transcription in vitro.. Virology.

[42] Jacob Y, Real E, Tordo N (2001). Functional interaction map of lyssavirus phosphoprotein: identification of the minimal transcription domains.. J Virol.

[43] Lo KW, Naisbitt S, Fan JS, Sheng M, Zhang M (2001). The 8-kDa dynein light chain binds to its targets via a conserved (K/R*X*TQT) motif.. J Biol Chem.

[44] Poisson N, Real E, Gaudin Y, Vaney MC, King S (2001). Molecular basis for the interaction between rabies virus phosphoprotein P and the dynein light chain LC8: dissociation of dynein-binding properties and transcriptional functionality of P.. J Gen Virol.

[45] Raux H, Flamand A, Blondel D (2000). Interaction of the rabies virus P protein with the LC8 dynein light chain.. J Virol.

[46] Tan GS, Preuss MAR, Williams JC, Schnell MJ (2007). The Dynein light chain 8 binding motif of rabies virus phosphoprotein promotes efficient viral transcription.. PNAS.

[47] Jayakar HR, Murti KG, Whitt MA (2000). Mutations in the PPPY motif of vesicular stomatitis virus matrix protein reduce virus budding by inhibiting a late step in virion release.. J Virol.

[48] Pornillos O, Alam SL, Rich RL, Myszka DG, Davis DR (2002). Structure and functional interactions of the Tsg101 UEV domain.. The EMBO Journal.

[49] Irie T, Harty RN (2005). L-Domain flanking sequences are important for host interactions and efficient budding of vesicular stomatitis virus recombinants.. J Virol.

[50] Gaudier M, Gaudin Y, Knossow M (2002). Crystal structure of vesicular stomatitis virus matrix protein.. The EMBO Journal.

[51] Roche S, Bressanelli S, Rey FA, Gaudin Y (2006). Crystal structure of the low p-H form of the VSV Glycoprotein G.. Science.

[52] Seif I, Coulon P, Rollin PE, Flamand A (1985). Rabies virulence: effect on pathogenicity and sequence characterization of rabies virus mutations affecting antigenic site III of the glycoprotein.. J Virol.

[53] Kongsuwan K, Cybinski DH, Cooper J, Walker PJ (1998). Location of neutralizing epitopes on the G protein of bovine ephemeral fever rhabdovirus.. J Gen Virol.

[54] Cybinski DH, Walker PJ, Byrne KA, Zakrzewski H (1990). Mapping of antigenic sites on the bovine ephemeral fever virus glycoprotein using monoclonal antibodies.. J Gen Virol.

[55] Lafon M, Wiktor TJ, Macfarlan RI (1983). Antigenic sites on the CVS rabies virus glycoprotein: analysis with monoclonal antibodies.. J Gen Virol.

[56] Prehaud C, Coulon P, Lafay F, Thiers C, Flamand A (1988). Antigenic site II of the rabies virus glycoprotein: structure and role in viral virulence.. J Virol.

[57] Bourhy H, Cowley JA, Larrous F, Holmes EC, Walker PJ (2005). Phylogenetic relationships among rhabdoviruses inferred using the L polymerase gene J Gen Virol.

[58] Tordo N, Poch, O, Ermine A, Keith G, Rougeon F (1986). Walking along the rabies genome: is the large G-L intergenic region a remnant gene?. Proc Natl Acad Sci USA.

[59] Tordo N, Poch O, Ermine A, Keith G, Rougeon F (1988). Completion of the rabies virus genome sequence determination: highly conserved domains among the L (polymerase) proteins of unsegmented negative-strand RNA viruses.. Virol.

[60] Poch O, Blumberg BM, Bougueleret L, Tordo N (1990). Sequence comparison of five polymerases (L proteins) of unsegmented negative-strand RNA viruses: theoretical assignment of functional domains.. J Gen Virol.

[61] Marston DA, McElhinney LM, Johnson NT, Muller KK, Conzelmann N (2007). Comparative analysis of the full genome sequence of European bat lyssavirus type 1 and type 2 with other lyssaviruses and evidence for a conserved transcription termination and polyadenylation motif in the G–L non-translated region.. J Gen Virol.

[62] Muller R, Poch O, Delarue M, Bishop DH, Bouloy M (1994). Rift Valley fever virus L segment: correction of the sequence and possible functional role of newly identified regions conserved in RNA-dependent polymerases.. J Gen Virol.

[63] Blumberg BM, Crowley JC, Silverman JI, Menonna J, Cook SD (1988). Measles virus L protein evidences elements of ancestral RNA polymerase.. Virology.

[64] Ogino T, Banerjee AK (2010). The HR motif in the RNA-dependent RNA polymerase L protein of Chandipura virus is required for unconventional mRNA-capping activity.. J Gen Virol.

[65] Nara T, Tanabe K, Mahakunkijcharoen Y, Osada Y, Matsumoto N (1997). The B cell epitope of paramyosin recognized by a protective monoclonal IgE antibody to Schistosoma japonicum.. Vaccine.

[66] Masters PS, Bhella RS, Butcher M, Patel B, Ghosh HP (1989). Structure and expression of the glycoprotein gene of Chandipura virus.. Virol.

[67] Masters PS, Banerjee AK (1987). Sequences of Chandipura virus N and NS genes: evidence for high mutability of the NS gene within vesiculoviruses.. Virol.

[68] Pattnaik AK, Ball LA, LeGrone A, Wertz GW (1995). The termini of VSV DI particle RNAs are sufficient to signal RNA encapsidation, replication, and budding to generate infectious particles.. Virol.

[69] Spiropoulou CF, Nichol ST (1993). A small highly basic protein is encoded in overlapping frame within the p gene of vesicular stomatitis virus.. J Virol.

[70] Chen H-L, Liu H, Liu Z-X, He J-Q, Gao L-Y (2009). Characterization of the complete genome sequence of pike fry rhabdovirus.. Arch Virol.

[71] Zhu RL, Lei XY, Ke F, Yuan XP, Zhang QY (2011). Genome of turbot rhabdovirus exhibits unusual non-coding regions and an additional ORF that could be expressed in fish cell.. Virus Research.

[72] Pinna LA (1990). Casein kinase 2: an ‘eminence grise’ in cellular regulation?. Biochim Biophys Acta.

[73] Basak S, Raha T, Chattopadhyay D, Majumder A, Shaila, MS (2003). Leader RNA binding ability of Chandipura virus P protein is regulated by its phosphorylation status: a possible role in genome transcription-replication switch.. Virology.

[74] Tesh R, Saidi S, Javadian E, Loh P, Nadim A (1977). Isfahan virus, a new vesiculovirus infecting humans, gerbils, and sandflies in Iran.. Am J Trop Med Hyg.

[75] Roche S, Rey FA, Gaudin Y, Bressanelli S (2007). Structure of the prefusion form of the vesicular stomatitis virus glycoprotein G.. Science.

[76] Venkateswarlu CH, Arankalle VA (2009). Recombinant glycoprotein based vaccine for Chandipura virus infection.. Vaccine.

[77] Schnell MJ, Conzelmann KK (1995). Polymerase activity of in vitro mutated rabies virus L protein.. Virol.

[78] Sleat DE, Banerjee AK (1993). Transcriptional activity and mutational analysis of recombinant vesicular stomatitis virus RNA polymerase.. J Virol.

[79] Bhattacharya R, Basak S, Chattopadhyay DJ (2006). Initiation of encapsidation as evidenced by deoxycholate-treated Nucleocapsid protein in the Chandipura virus life cycle.. Virology.

[80] Rodriguez LL, Pauszek SJ, Bunch TA, Schumann KR (2002). Full-length genome analysis of natural isolates of vesicular stomatitis virus (Indiana 1 serotype) from North, Central and South America.. Jr Gen Virol.

[81] Whelan SPJ, Wertz GW (1999). Regulation of RNA Synthesis by the Genomic Termini of Vesicular Stomatitis Virus: Identification of Distinct Sequences Essential for Transcription but Not Replication.. JVirol.

[82] Stillman EA, Whitt MA (1998). The length and sequence composition of vesicular stomatis virus intergenic regions affect mRNA levels and the site of transcription initiation.. J Virol.

[83] Barr JN, Whelan SPJ, Wertz GW (1997). Role of the intergenic dinucleotide in vesicular stomatitis virus RNA transcription.. J Virol.

[84] Nichol ST, Holland JJ (1987). Genomic RNA terminus conservation and diversity among Vesiculoviruses.. J Virol.

