# Supplementary figures and images for: Whole Genomes of Chandipura Virus Isolates and Comparative Analysis with Other Rhabdoviruses
Source: PLoS One. 2012 Jan 17;7(1):e30315. doi: 10.1371/journal.pone.0030315 (PMC3260278; doi:10.1371/journal.pone.0030315)

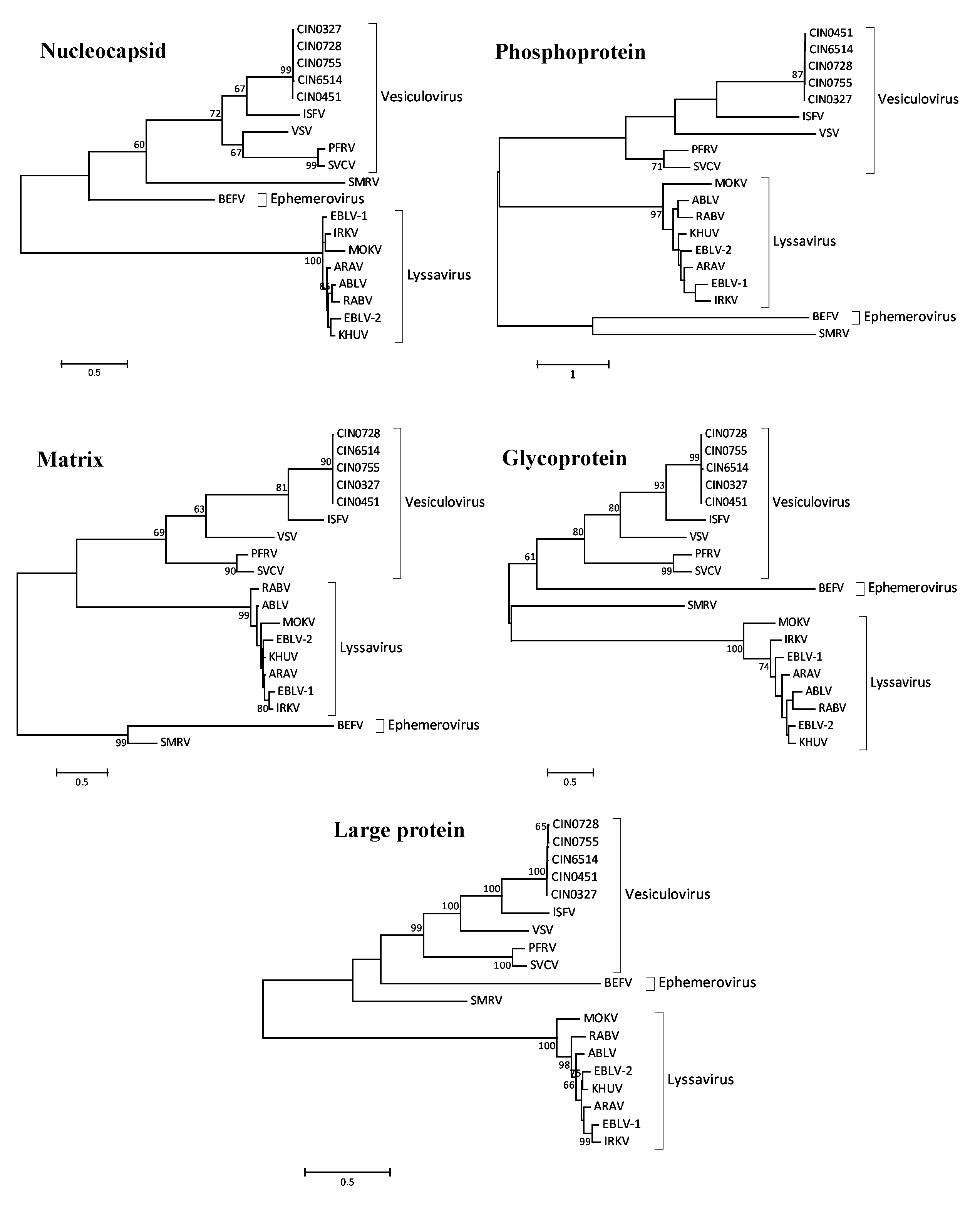

Supplement: Figure S1 — (TIF) [file pone.0030315.s001.tif]

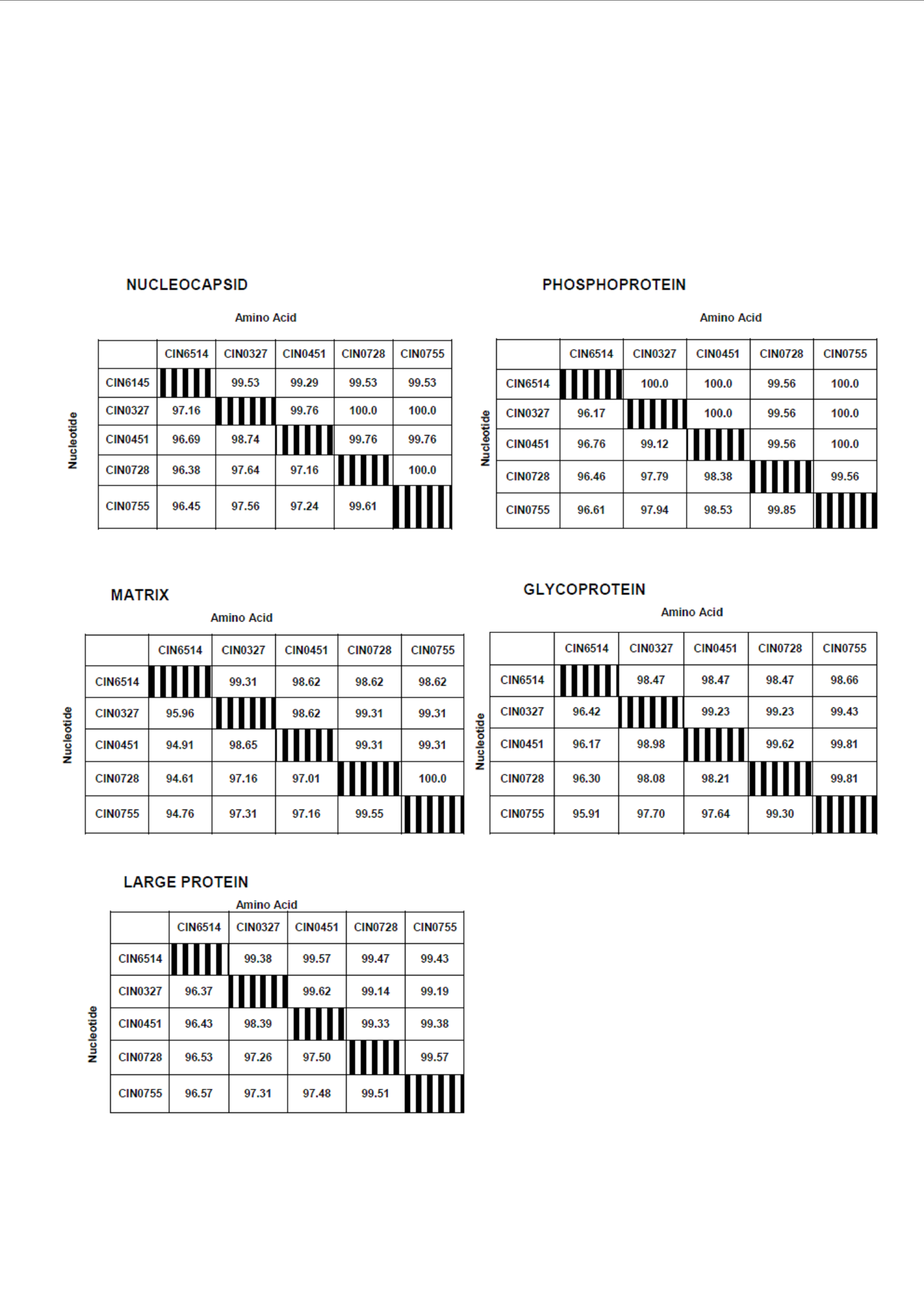

Supplement: Figure S2 — (TIF) [file pone.0030315.s002.tif]

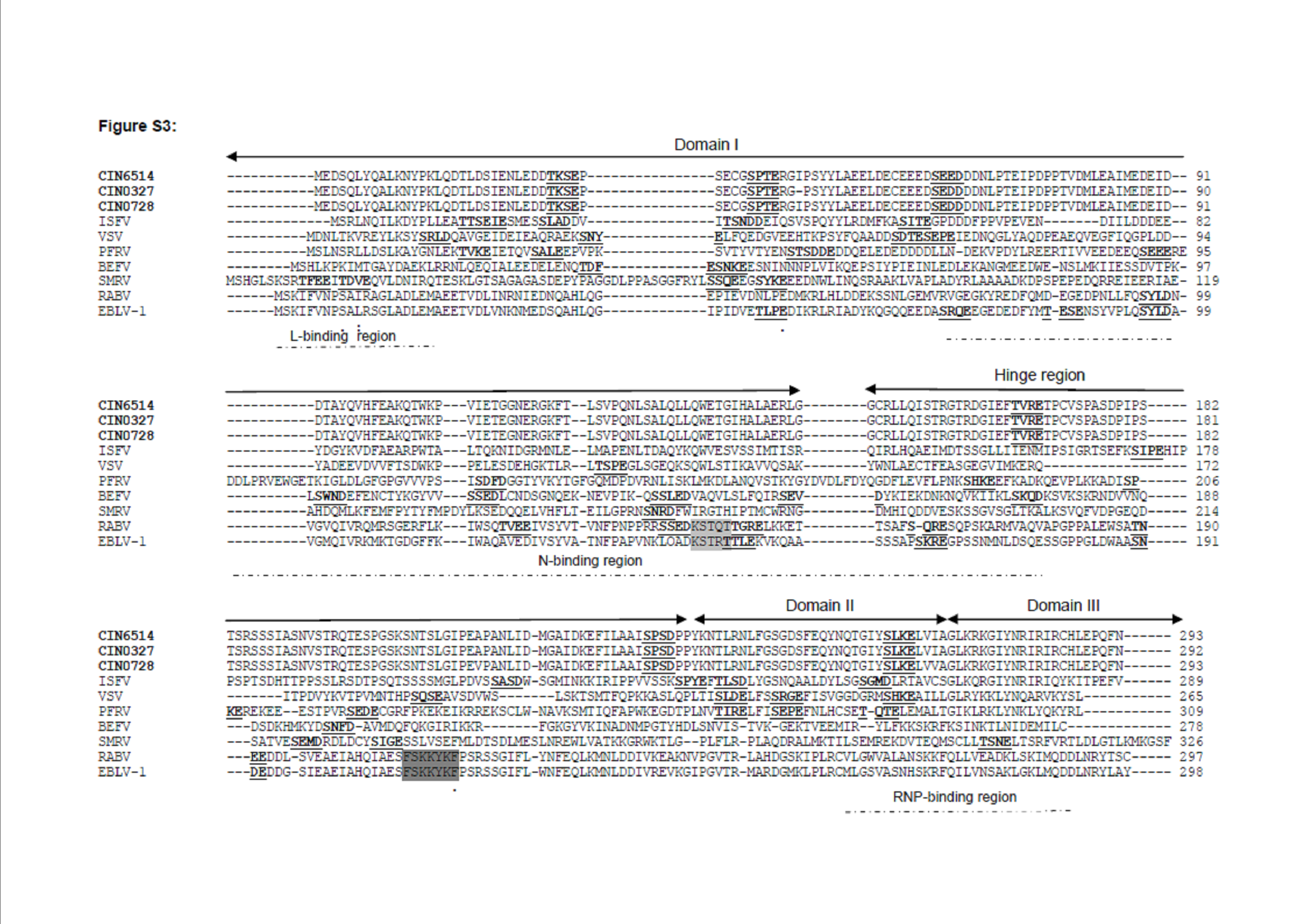

Supplement: Figure S3 — (TIF) [file pone.0030315.s003.tif]

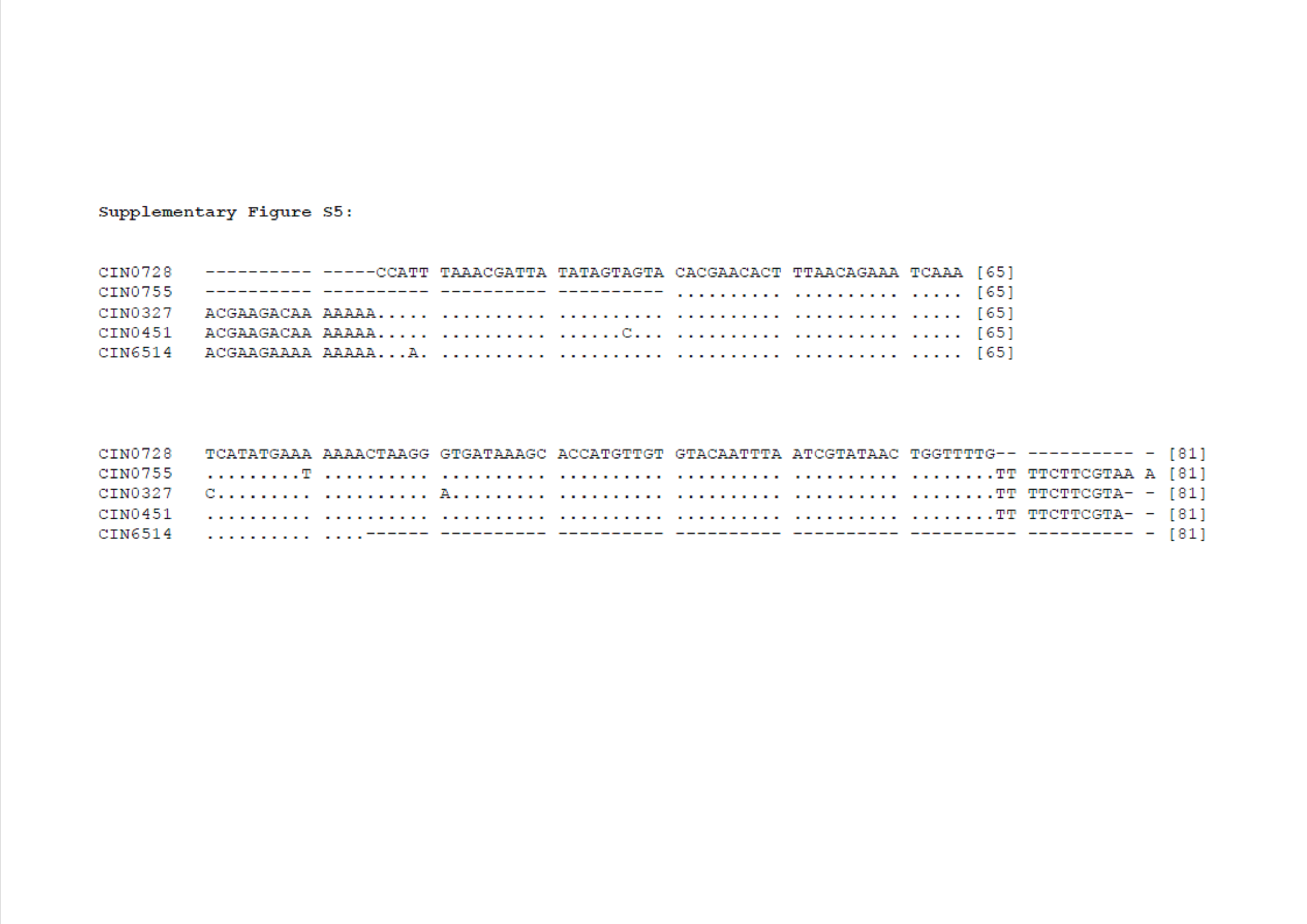

Supplement: Figure S5 — (TIF) [file pone.0030315.s005.tif]
