# Supplementary material for: Whole Genomes of Chandipura Virus Isolates and Comparative Analysis with Other Rhabdoviruses
Source: PLoS One. 2012 Jan 17;7(1):e30315. doi: 10.1371/journal.pone.0030315 (PMC3260278; doi:10.1371/journal.pone.0030315)
Supplement: Figure S4 — (PDF) [file pone.0030315.s004.pdf]

Figure S4:

```
CIN6514             --------MDLNPVDDAAE--LSEENFFSGKLSK-ECRIRGLNSVDYNLNSPLVSDDLTYLLDKFKGKPVPIRWK--------MKKWDSILDQLRKHDLEYLRP--SDLHQWFAEWMLYS 99
CIN0327             --------MDLNPVDDAAE--LSEENFFSGKLSK-ECRIRGLNSVDYNLNSPLVSDDLTYLLDKFKGKPVPIRWK--------MKKWDSILDQLRKHDLEYLRP--SDLHQWFAEWMLYS 99
CIN0728             --------MDLNPVDDAAE--LSEENFFSGKLSK-ECRIRGLNSVDYNLNSPLVSDDLTYLLDKFKGKPVPIRWK--------MKKWDSILDQLRKHDLEYLRP--SDLHQWFAEWMLYS 99
ISFV                --------MDEYSEEKWGD--SDEESFGTGKYSD-ESRIRGLNSVDYNLNSPLIQDDLYYLMERVRGRPVPPIWK--------AKNWTETIHLVQESRLDYLPT--QKLHSWYAEWLMEE 99
VSV                 ------MEVHDFETDEFND--FNEDDYATREFLNPDERMTYLNHADYNLNSPLISDDIDNLIRKFNSLPIPSMWD--------SKNWDGVLEMLTSCQANPIST--SQMHKWMGSWLMSD 102
PFRV                --------MFDWESQDVPSGFPDEESYFPTSKLSVEERMHYLNNVDYNLNSPLISDDIEYLTMKHFGRSIPALWK--------AKNWEIPLDMLKD-VSKIRSW--DQIHPWMGKWFNS- 100
SMRV                --------MDGTEEIKDSVPLWMETDELESEEKQ--EWMEFINNVDYNLNSPLIRDEVDSFVKYLNTDNLESRFAR-------FRQWKIDKKCIKELLPSAPRW--VGSSGSQRIWSYLC 101
BEFV                MDSVDFLDSEDHDQNSWNGEEFGDDFLEYIWDQDEEESMDLINNKDYNLNSPLIIDPLVELRNWINNEKGHSESDLRSQQSFEFKEFDIIKRVLRNFINNVNLRRPEDSHHIFAKFLNQA 120
EBLV-1              ----------MIDSTEVYDDPVDPVEPEADLRS-NSVVPNILRNSDYNLNSPLLEDPSRLMIEWLTTGNRPSRLNLTDN---LLRSYKVLRGYFKKLDVGSMRAGGLGAQAMITLWLHGS 106
KHUV                ----------MIDSLEVYDDPVDPIEPEVDLKN-NAVVPNILRNSDYNLNSPLIEDPSQLMLDWLITGNKPNRLSPSDN---LVRSYKVLKSYFKKVDVGSLRIGGLGAQAMTSLWLHGA 106
RABV                ----------MLDPGEVYDDPIDPIESEAEPRG-TPTVPNILRNSDYNLNSPLIEDPAKLMLEWLKTGNRPYRMTLTDN---CSRSYKVLKDYFKKVDLGSLKVGGTAAQSMVSLWLCGA 106
                                   .                         :.  ********: *    :                       :.:      . .                   :

CIN6514             KHGSKQ---GEDFLKTVDEEASDTFEVVRSFIRG---WTGGE---INFVRKS--GKHMGYCAELCQKFLDLHKLTLLGNAATDNELLQLS-----KTFG-----DDKIYKKRLIKLPSLG 198
CIN0327             KHGSKQ---GEVFLKTVDEEASDTFEVVRSFIRG---WTGGE---INFIRKS--GKHMGYCAELCQKFLDLHKLTLLGNAATDNELLQLS-----KTFG-----DDKIYKKRLIKLPSLG 198
CIN0728             KHGSKQ---GEDFLKTVDDEASDTFEVVRSFIRG---WTGGE---VNFVRKS--GKHMGYCAELCQKFLDLHKLTLLGNAATDNELLQLS-----KTFG-----DDKIYKKRLIKLPSLG 198
ISFV                SHDSSQ---GLAFLKEVDKDSLETYEVVMSFLRG---WCGGA---PAYKKKE--GRHIAKIGSLCQKFLDLHRVILIMNASTQMELSNLA-----ETFQ-----ASSVSKK-IITTPSMG 197
VSV                 NHDASQ---GYSFLHEVDKEAEITFDVVETFIRG---WGNKP---IEYIKKERWTDSFKILAYLCQKFLDLHKLTLILNAVSEVELLNLA-----RTFKGKVRRSSHGTNICRIRVPSLG 208
PFRV                EHKSPQ---GESFLRTVQAESELTAEIPITFLKG---WIGKD---IPYPVKR----GHGAVNTLMQKVLDLHKLTLLINSVDVTETKRLC-----ESFG-------IDPKFSRFNTHSLG 195
SMRV                KSNTSARKYSHILLKQTQRESNEISGIQQAFFKG---WIGKD---IGPVEKK----HPVEVTKWLEIFLEFHIMVLMLNAKSKSERDNLC-----TTFG---FALSEDRRSAGWDSQFLG 203
BEFV                IVTNDY----WNLGETLSKIVEDIREVGASFYRG---LDLDENIIIDNIRRN-WNDVPQLGKSWFLKFFELHRVICVMNARSNIELKNLQSKNKLKKIKLPKELEERGHKCWIFDLDISG 232
EBLV-1              HSESTRSRKCLTELSQFYKKSSPIEKLLNYTLENRGLKTPTEGVLSSINKVQYDQAFGRYLGNTYSSYLFFHVIILYMNALDWDEEKTILA--LWKEIAS----IDVKSDLVKFRDQIWG 220
KHUV                HSESTRSRKCLSDLAQFYQKSSPIEKLLNYTLENRGLQVPPEGVLSSLKKVSYDRAFGRYLANTYSSYLFFHVIILYMNALDWEEEKTILA--LWKDLNS----VDIKKDQVKFRDQIWG 220
RABV                HSESNRSRRCITDLAHFYSKSSPIEKLLNCTLGNRGLRIPPEGVLNCLERVNYDKAFGRYLANTYSSYLFFHVITLYMNALDWEEEKTILA--LWKDITS----VDTEKDLVKFKDQIWG 220
                                              :      .                                  : :* :    *:    *   :        :                     *

CIN6514             RVIFDSGFFIVLDQRVLMDRNFMLMMKDVIIGRMQTVLSMISRCDDKFSS--KDIDFLLKVYSTGDKIIRKLGNDGYELIKTVEPMCNLRLSDLARRFRPLIPPFPHFRRHIESTVDEL- 315
CIN0327             RVIFDSGFFIVLDQRVLMDRNFMLMMKDVIIGRMQTVLSMISRCDDKFSS--KDIDFLLKVYSTGDKIIRKLGNDGYELIKTVEPMCNLRLSDLARRFRPLIPPFPHFRRHIESTVDEL- 315
CIN0728             RVIFDSGFFIILDQRVLMDRNFMLMMKDVIIGRMQTVLSMISRCDDKFSS--KDIDFLLKVYSTGDKIIRKLGNDGYELIKTVEPMCNLRLSDLARRFRPLIPPFPHFRRHIESTVDEL- 315
ISFV                KMEMSGQFALAYQQKVILDRNFLLMMKDVVIGRMQTLLSMVSRTDDKFSD--GDISYLIKIYQLGDKIIQSLGNDGYELIKTIEPMCNLRLSDLAREYRPLIPEFPHFRQHIEGTVSEL- 314
VSV                 PTFISEGWAYFKKLDILMDRNFLLMVKDVIIGRMQTVLSMVCRIDNLFSE--QDIFSLLNIYRIGDKIVERQGNFSYDLIKMVEPICNLKLMKLARESRPLVPQFPHFENHIKTSVDEG- 325
PFRV                VVRYCPNWIFIEDAAVLLDRNFLLMMKDTLIGRLQTLVSMLGNYEMTEDQTYQHTDTMLSLYSYGDQIISKSGNEGYSKIKLLEPICNLRLSELAHTYRPLVPEFPHFKDHVETSVREE- 314
SMRV                PIIITGSWILIVNRHTVMERNYALMIKDTCVGRFMTLSSMIGRFDEMFTD--EHLRSVLHFYALGDEYVSLNGNEAYSGIKLVEPMCNDRLAELAAEFRPLIPKFTNFTEHVRTAVEEA- 320
BEFV                RWIIFDNYAYWELQRIVLNREFILMMKDVLISRFQTVLSMNVCTDEYKYT-EENIETMMSLYREGDLILEEHGNKSYKGLKLLESICNLRLIKIVRKSRPKIPEFPNFENHIYSSLNDLR 351
EBLV-1              SLVITKDFVYSQSANCLFDRNYTLMLKDLFLSRFNSLLILISPPESRYSD--DLVSNLCQLYIAGDKVLSACGNSGYDVIKLLEPYIVNKLVQKAEKFRPLIHSLGDFPQFIRDKTNQLE 338
KHUV                SLVVTKDFVYSQSANCLFDRNYTLMLKDLFLSRFNSLLILLSPPEPRYSD--DLISQLCQLYIAGDNVLSMCGNSGYDVIKMLEPYVVNSLVQRAEGFRPLIHSLGDFPLFIKDKVTQLE 338
RABV                LLIVTKDFVYSQSSNCLFDRNYTLMLKDLFLSRFNSLMILLSPPEPRYSD--DLISQLCQLYIAGDQVLSMCGNSGYEVIKILEPYVVNSLVQRAEKFRPLIHPLGDFPMFIKDKVNQLE 338
                           :        .::*:: **:**  :.*: :: :  :    :            :   *  **  :   ** .*. :* :*.     * . .   ** :  :.*  ::    :
          I

CIN6514             SAKTPLIRELFSLIDTSPNVDSTLVVYGSFRHWGHPFINYFEGLEKLHKQVTMEKEIDTNYSEALASDLARIVLTKEFNEKKQWAVDYHRVPTNHPFKNHIRDNTWPTAAVIQDFGDHWH 435
CIN0327             SAKTPLIRELFSLIDTSPNVDSTLVVYGSFRHWGHPFINYFEGLEKLHKQVTMEKEIDTNYSEALASDLARIVLTKEFNEKKQWAVDYHRVPTSHPFKNHIRDNTWPTAAVIQDFGDHWH 435
CIN0728             SAKTPLIRELFSLIDTSPNVDSTLVVYGSFRHWGHPFINYFEGLEKLHKQVTMEKEIDTNYSEALASDLARIVLTKEFNEKKQWAVDYHRVPANHPFKNHIRDNTWPTAAVIQDFGDHWH 435
ISFV                RKKTALIVDMFKMIDRTPGVDITLVIYGSFRHWGHPFIDYFAGLTKLNSQVTMGKQIDDEYVACLASDLARIVLTKEFNEKKRWSVNYNLVPQDHPFHEHIRDNTWPTPAVIQDFGDKWH 434
VSV                 AKIDRGIRFLHDQIMSVKTVDLTLVIYGSFRHWGHPFIDYYTGLEKLHSQVTMKKDIDVSYAKALASDLARIVLFQQFNDHKKWFVNGDLLPHDHPFKSHVKENTWPTAAQVQDFGDKWH 445
PFRV                DTTDGLLSSIFQLVKDTDDIQLILTIYGSFRHWGHPFISYFEGLRKLHSQVTLPKIIDREYAAALASDLAYTVLQRKFSEEKKWYVDADELPDRHPLKEHIENGTWPTAAQIQDFGDRWN 434
SMRV                ADPGGHLHMLHGLIKRETDVNLLLIYYGSFRHWGHPYVEYAEGMEKLHRQVTMKKDVDDKMAQCLASDLAYMVLKKQFDSKRKWMYNKEALPDKHPFKEHIADSAWPTPAQIEQFGDNWH 440
BEFV                VERGIDLSKFSNIILREESIDMVLAFYSSFRHFGHPWIDYLTGLDKLESQVNKDCQVDIQYANLLASDLAFKILRKNFLEKKCWSYDKNKMDKKHKLYHHISHNTWPTQQIIDEFGDHWH 471
EBLV-1              GTFGPSAREFFQTMDLLDNIHDLVFVYGCYRHWGHPYIDYRKGLSKLYDQVHVKKIIDRNYQECLASDLAKRILRWGFDKYSRWYLDSNLLPGDHPLSPYVKTQTWPPKHVVDMVGDTWH 458
KHUV                GTFGPSARNFFRILDQLDNIHDLVFVYGCYRHWGHPYIEYRKGLSKLYDQVHVKKVIDGDYQECLASDLAKRILRWGFDKYSKWYLDSKLLAPNHPLTPYIKTQTWPPKHIVDMVGNTWH 458
RABV                GTFGPSAKRFFRVLDQFDNIHDLVFVYGCYRHWGHPYIDYRKGLSKLYDQVHIKKVIDKSYQECLASDLARRILRWGFDKYSKWYLDSRFLARDHPLTPYVKTQTWPPKHIVDLVGDTWH 458
```

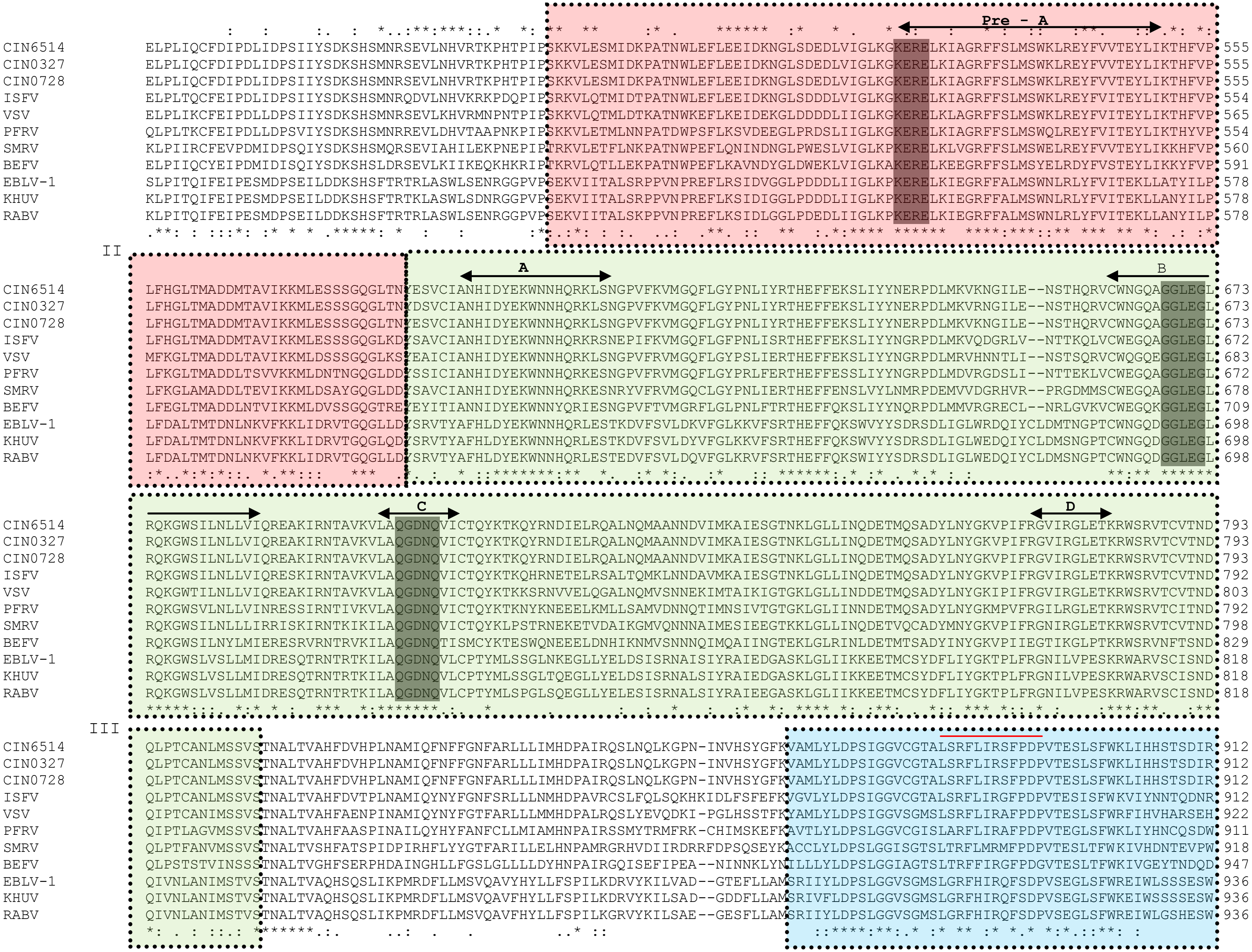

Pre - A
A
B
C
D
II
III
CIN6514
CIN0327
CIN0728
ISFV
VSV
PFRV
SMRV
BEFV
EBLV-1
KHUV
RABV

```
CIN6514   LKNLSEQFGNPKIAVFRESHIEKLLEDPTSLNISMGMSAANLLKTEIKKNLLQKKSSIGNQIVKDAVYYIHSEDEKLRTFLWSITPLFPRFLSEFKAGTFMGVASSIVSLFQNSRTIRNV 1032
CIN0327   LKNLSEQFGNPKIAVFRESHIEKLLEDPTSLNISMGMSAANLLKTEIKKNLLQKKSSIGNQIVKDAVYYIHSEDEKLRTFLWSITPLFPRFLSEFKAGTFMGVASSIVSLFQNSRTIRNV 1032
CIN0728   LKNLSEQFGNPRIAVFRESHIEKLLEDPTSLNISMGMSAANLLKTEIKKNLLQKKSSIGNQIVKDAVYYIHSEDEKLRTFLWSITPLFPRFLSEFKAGTFMGVASSIVSLFQNSRTIRNV 1032
ISFV      LKKLCTAFGNPKIAQFRYSHIEKLLEDPTSLNISMGMSAANLLKSEIKKNLLRKRRTIGNSIVRDAVTYIHSEDEKIRSYLWSINPLFPRFLSEFKSGTFMGVASSVVSLFQNSRTIRNV 1032
VSV       LKEMSAVFGNPEIAKFRITHIDKLVEDPTSLNIAMGMSPANLLKTEVKKCLIESRQTIRNQVIKDATIYLYHEEDRLRSFLWSINPLFPRFLSEFKSGTFLGVADGLISLFQNSRTIRNS 1042
PFRV      LKKLAKRCGNPKLAKFRPEHIPKIIEDPAALNISMGMSASNLLKTEVKGHLIRTADSIQNQIIREAAEYLGQEEESLNEFLWDIEPFFPRFLSEFRSSTFVGVTDSLIGLFQNSKTIRGL 1031
SMRV      IRKLSVGAGNPRLSPGSSADLSKLIEDPTSLNIPKGISALTIMKEEVKKNLYRISHKLENHMARDAIDYSRDEEEQMMLFLSSIRPLFPRFLSEFKNATYLGITDSLIALFQNSRTIRNL 1038
BEFV      IKRLASTVGSPELSPFKPEDLDKLIEKPESLNIKHGLSSSNMIKGEVKKNIIENCSKIQNEIIRDAARNLVSEENQLFLWLRTINPLFPRFLSQFAESTYYGVTKSLINLFTNSKTIRGI 1067
EBLV-1    IHALCQEAGNPDLGDRSLESFTRLLEDPTTLNIRGGASPTILLREAIRKALYDEVDKVENSEFREAILLSKTHRDNFILFLRSVEPLFPRFLSELFSSSFLGIPESIIGLIQNSRTIRRQ 1056
KHUV      IHSLCQEAGNPDLGDRSLESFTRLLEDPTTLNIRGGASPTILLKEAIRKALYDEVDKVENSEFREAILLSKTHRDNFILFLRSIEPLFPRFLSELFSSSFLGIPESIIGLIQNSRTIRRQ 1056
RABV      IHALCQEAGNPDLGERTLESFTRLLEDPTTLNIKGGASPTILLKDAIRKALYDEVDKVENSEFREAILLSKTHRDNFILFLKSVEPLFPRFLSELFSSSFLGIPESIIGLIQNSRTIRRQ 1056
          :: :.   *.* :.      : :::*.* :***  * *.  :::  ::  :      .: *    ::*       . : :  :*  : *:******::  .:: *:...:: *: **:***

IV
CIN6514   FRDYMSQTIDDLIVKSELTSLEHLSNYTD--RKGSGGIWGCSAEQADKLRRMSWKRPVLGTTVPHPLEMHGRGTLKSPLSKCCK--ESRMDYISVHIPEGLNKVLDGRGSLPAYLGSKTS 1148
CIN0327   FRDYMSQTIDDLIVKSELTSLEHLSNYTD--RKGSGGIWSCSAEQADKLRRMSWKRPVLGTTVPHPLEMHGRGTLKSPLSKCCK--ESRMDYISVHIPEGLNKVLDGRGSLPAYLGSKTS 1148
CIN0728   FRDYMSQTIDDLIVKSELTSLEHLSNYTD--RKGSGGIWGCSAEQADKLRRMSWKRPVLGTTVPHPLEMHGRGTLKSPLSKCCK--ESRMDYISVHIPEGLNKVLDGRGSLPAYLGSKTS 1148
ISFV      FKDYMSSAIDELITKSEVNSLEHLCKYKG--VRNFDQVWKCSASQADYLRRLSWGRKVLGTTIPHPLEMLGAGTIKNNSSTCCE--HSGQDYISVFCPKGISNVLIERGPMAAYLGSKTS 1148
VSV       FKKKYHRELDDLIVRSEVSSLTHLGKLHL--RRGSCKMWTCSATHADTLRYKSWGRTVIGTTVPHPLEMLGPQHRKETPCAPCN--TSGFNYVSVHCPDGIHDVFSSRGPLPAYLGSKTS 1158
PFRV      FRSFYKRELDRLVVKSELSSLEHLGSYR---KETPDAIWDCSSLQADLLREKSWGRSVIGMTVPHPLEMFGTGHLKEEECIPCQ--TSGLTYISSYCPKGINNWYCTVGSLAAYLGSKTS 1146
SMRV      FTSKYKREIDHKILKGEITSMVRLCTIER--QRLGNDIWQCSASQADRLRSESWGSQVVGTTVPHPAELLGTPSLSSATCEGCLGPNDRRGYITVSVPRGLAGYLDSRGPLMAYLGSKTS 1156
BEFV      YKKKYRKELDQLMIKGEVRSIFGLIKIVNRSKQFVMPIWDCSASLADSLRKRSWGKEVLGTTVPHPAEMFKGYRGGEDSCSFCRGNGSNNNYLTVLMPRGIPMKCHYRGPYYYPYLGSNTK 1187
EBLV-1    FRRSLSRTLEESFFNSEIHGISRMTQVPQ----RIGRVWSCSAERADQLREISWGRKVVGTTVPHPSEMLALIPKSSISCTCGQ-SGDDSPRISVSVLPSLDQSFFSRGPLKGYLGSSTS 1171
KHUV      FRKSLSRTLEESFFNSEVHGINRMTQVPQ----RIGRVWPCSAERADLLREISWGRKVVGTTVPHPSEMLALLPKSSISCVCGQ-TGEDSPRISVSVLPSFDQSFFSRGPLKGYLGSSTS 1171
RABV      FRKSLSRTLEESFYNSEIHGINRITQTPQ----RVGRVWPCSSERADLLREISWGRKVVGTTVPHPSEMLELFPKSSISCTCGA-TGGGNPRVSVSVLPSFDQSFFSRGPLKGYLGSSTS 1171
           :       ::  .  ..*:  .:  :          :* **:   ** **   **    *:* *:*** *:           .  .           ::      .:        *.    ****.*.

CIN6514   ESTSILQPWEKESKIPIIRRATRLRDAIHWFVDPDSNLARSILNNIESLTGEKW-EGALKGYKRTGSALHRFSTSRVSHGGFSSQSPACLTRMMATTDTMRDYAQ--LNYDFMFQASLLY 1265
CIN0327   ESTSILQPWEKESKIPIIRRATRLRDAIHWFVDPDSNLARSILNNIESLTGEKW-EGALKGYKRTGSALHRFSTSRVSHGGFSSQSPACLTRMMATTDTMRDYAQ--LNYDFMFQASLLY 1265
CIN0728   ESTSILQPWEKESKIPIIRRATRLRDAIHWFVDPDSNLARSILNNIESLTGEKW-EGALKGYKRTGSALHRFSTSRVSHGGFSSQSPACLTRMMATTDTMRDYAQ--LNYDFMFQASLLY 1265
ISFV      ESTSILQPWEKESKIPIIKRATRLRDAIHWFVEPSSNLAKSILQNITALTGEEW-GSSLEGFKRTGSALHRFTTSRMSHGGFCAQSPAALTRMMATTDTMSDYAK--DNYDFMFQACLLF 1265
VSV       ESTSILQPWERESKVPLIKRATRLRDAISWFVEPDSKLAMTILSNIHSLTGEEW-TKRQHGFKRTGSALHRFSTSRMSHGGFASQSTAALTRLMATTDTMRDLGD--QNFDFLFQATLLY 1275
PFRV      ETTSILQPWEKDSKVPIIKRATKLRDSISWFVQPDSKLAKSIQQNLKALTGEDW-EEDIQGFKRTGSALHRFTTSRISNGGFSAQSPAKLTRIMTTTDTMRDLGD--QNYDFMFQAGILY 1263
SMRV      ETTSLLQPWERETKIPLIKRAAKLRTPISWFVQPDSNLAASIMGNLESLTGENW-SNAIAGFKRTGSALHRFSCSRVSAGGYSGQSPAKLTRMVSTTDTFMAPTD--DNYDFMFQSLLIH 1273
BEFV      ESTSILQPWEKETKVPVLKRACDLRKSINWFVTPDSLLAKSIFNNLKALTGEDW-EDQIKGYKRTGSSLHRFGCSRVSSGGFSASSPSCFTWCIATTDTMCGLGE--VNYDFMFQSTLVW 1304
EBLV-1    MSTQLFHAWEKVTNVHVVKRALSLKESINWFITRDSNLAQTLIRNIVSLTGPFFPLEEAPVFKRTGSALHRFKSARYSEGGYSSICPNLLSHISVSTDTMSDLTQDGTNFDFMFQPLMLY 1291
KHUV      MSTQLFHAWEKVTNVHVVKRALSLKESINWFIVRDSNLAQVLIKNIISLTGPLFPLEETPVFKRTGSALHRFKSARYSEGGYSSICPNLLSHISVSTDTMSELTQDGKNFDFMFQPLMLY 1291
RABV      MSTQLFHAWEKVTNVHVVKRALSLKESINWFINRNSNLAQTLIGNIMSLTGPDFPLEEAPVFKRTGSALHRFKSARYSEGGYSSVCPNLLSHISVSTDTMSDLTQNGKNYDFMFQPLMLY 1291
            .*.:.:. **: .:. .::**  *:  * **:    * **  :  *: :***  :        :*****:****  :* * **:        ::      :***:          *:**:**  ::

V
CIN6514   SQMTSSVILMGT--TVSNTIHFHVTCRKCIREITEPMLESPREYRGKDVHLVLAKWKN-SSNGWGETLQLLKPVEGD--WDTIPPVEKSYHVGRILGFLYGDLKSQNSSRADDSSIFPLS 1380
CIN0327   SQMTSSVILMGT--TVSNTIHFHVACRKCIREITEPMLESPREYRGKDVHLVLAKWKN-SSNGWGETLQLLKPVEGD--WDTIPPVEKSYHVGRILGFLYGDLKSQNSSRADDSSIFPLS 1380
CIN0728   SQMTSSVILMGT--TVSNTIHFHVTCRKCIREITEPMLESPREYRGKDVHLVLAKWKN-SSSGWGETLQLLKPVEGD--WDTIPPVEKSYHVGRILGFLYGDLKSQNSSRADDSSIFPLS 1380
ISFV      SQITTSVLLLET--TISNTVHFHTRCINCVRKIEEPWLESPSVLQSKDVSNVLASWRN-GGGSWGEQLHQLKPLKGD--WEILTPAEKSYHVGRTLGFLFGDLTGQSSIRADDSSLFPLS 1380
VSV       AQITTTVARDGW--ITSCTDHYHIACKSCLRPIEEITLDSSMDYTPPDVSHVLKTWRN-GEGSWGQEIKQIYPLEGN--WKNLAPAEQSYQVGRCIGFLYGDLAYRKSTHAEDSSLFPLS 1390
PFRV      SQMTTGEMREGS--VNSTATHYHISCKSCLREIQEPMLESRVIYNPPSSSRIIRSWVP-GTAGMMEESKSMVLREVD--WDPLTRHEKSYHIGRCQGFLYGDLTYQKTGRSEESSIFPLS 1378
SMRV      SQITVGEIHDGV--KENAVYHQHISCTGCLRKIDEPYLESEWVYNFQPVHTQLSQWRG-GQGGWSTASEPIVLQPAD--WTKMSKRLQSFHVGRSIGFMYGDLTCISSSQKDDSSLFPLS 1388
BEFV      CQMSSIIRERGN--LHSKIHHYHIKCNKCLREIQEPVLESGWEYQPRNVSQILEKWRPKNMKTWGEEKIHMDIKDNDDEWDNLTVEDKSYEIGKTIGWLVGDSLLSHKRNYEFKSLFPVS 1422
EBLV-1    AQTWTSELVQKDLRLRDSTFHWHLRCLKCIRPIDDITLEAPKIFSFPDVSKRISRMVS-GAVPQFRKLPEIGLKPGK--FDSLKEKDKSRHIGTAQGLLYSILVATHDSGYNDGTIFPVN 1408
KHUV      AQTWTSELVQKDLRLKDSTFHWHLRCPKCIRPIDDITLEAPQVFMFPDVSKRISRMVS-GAVPQFQRLPEISLKPGK--FESLNGKDKSRHIGTAQGLLYSILVATHDSGYNDGTIFPVN 1408
RABV      AQTWTSELVQRDTRLRDSTFHWHLRCNRCVRPIDDITLETSQIFEFPDVSKRISRMVS-GAVPQFQKLPDIRLRPGD--FESLSGREKSRHIGSAQGLLYSILVAIHDSGYNDGTIFPVN 1408
           .*                .    * *   *  *:* *   *::               :       .          :      .  :   :     :* .:*    * : .            :  ::**:.
```

```
CIN6514         IQMRLRGRGFLRGILDGLVRASACQVIHRRSVALLSKPANAIYGGLIYLIDKISASTSFTTLCRDGPIREELSSIPHKIPTSYPTSNSDMGLHIRNYLKFQCKTVELG---KYQSDIKDL 1497
CIN0327         IQMRLRGRGFLRGILDGLVRASACQVIHRRSVALLSKPANAIYGGLIYLIDKISASTSFTTLCRDGPIREELSSIPHKIPTSYPTSNSDMGLHIRNYLKFQCKTVELG---KYQSDIKDL 1497
CIN0728         IQMRLRGRGFLRGILDGLVRASACQVIHRRSVALLSKPANAIYGGLIYLIDKISASTSFTTLCRDGPIREELSSIPHKIPTSYPTSNSDMGLHIRNYLKFQCKTVELG---KYQSDIKDL 1497
ISFV            IQKRLRGRGFLRGVLDGLVRASACQVIHRRSLTQLKRPANAVYGGLIFLIDKISASSTFINLCRDGPIREELSSIPHKIPTSYPTSNADLGLHIRNYFKFQCKSVELG---KYQSDLEDL 1497
VSV             IQGRIRGRGFLKGLLDGLMRASCCQVIHRRSLAHLKRPANAVYGGLIYLIDKLSVSPPFLSLTRSGPIRDELETIPHKIPTSYPTSNRDMGVIVRNYFKYQCRLIEKG---KYRSHYSQL 1507
PFRV            IQYKVEGSGFLKGFCDGIIRASAVQALHRR-ISSIVSTADVIYGGALYLTNQVGDSPPFQNLCRAGPLREELERIPHKMTSSYPTSNSDMGYLIRNYLKRSLKQLSRG---RYETKEGTL 1494
SMRV            IQRKLIPEAFFEGILEGLVRAASLDLINRRSVRKLSRPYVAILGTVGYLIGELSSSAGMRNLTRKGPLSSELTTVPHKVPPSYPMTDLDLGALLRNYLQTKFRHNYDGGWQNYTSLDTRI 1508
BEFV            IRYKLEGLPFLEGILDGFKICGSLNLTHRRNYMILKKPKLALQGTVFFLIDRSSFISEFTNFISHPKIYKAIKSLPHKIPTSYPMNLSDLGSILRSFLKQLYYRRKEL------IIKKSS 1536
EBLV-1          IYGKVSPRDYLRGLARGVLIGSSICFLTRMTNININRPLELISGVISYILLRLDNHPSLYVMLKEPSLRSEIFSIPQKIPAAYPTTMKEGNRSVLCYLQHVLRYEREA--ITTSPENDWL 1526
KHUV            IYGKVSPKDYLRGLARGVLIGSSICFLTRMTNININRPLELISGVISYILLRLDNHPSLYVMLKEPSLRSEIFSIPQKIPAAYPTTMKEGNRSVLCYLQHVLRYERDV--ITASPENDWL 1526
RABV            IYGKVSPRDYLRGLARGILIGSSICFLTRMTNININRPLELISGVISYILLRLDNHPSLYIMLREPSLRGEIFSIPQKIPAAYPTTMREGNRSILCYLQHVLRYEREA--ITASPENDWL 1526
                *  ::    ::.*.   *.    ..      *      :  .    : *    ::  .  .   . :  :       :    :   :*:*:..:** .   :  .   :   :::

CIN6514         WLFSDVM--TSNIAGPFALSTKILKCLYKPALSQKDRNNIRKISNFSKMMRSQL------SWDPTS--SEFITSQILVCNEEIRHACKFGIPK-------LSLK-FDDPVWGPEDYGLIW 1599
CIN0327         WLFSDVM--TSNIAGPFALSTKILKCLYKPALSQKDRNNIRKISNFSKMMRSQL------NWDPTS--SEFITSQILVCNEEIRHACKFGIPK-------LSLK-FDDPVWGPEDYGLIW 1599
CIN0728         WLFSDVM--TSNIAGPFALSTKILKCLYKPALSQKDRNNIRKISNFSKMMRSQL------SWDPTS--SEFITSQILVCNEEIRHACKFGIPK-------LSLK-FDDPVWGPEDYGLIW 1599
ISFV            WLFSDLL--SSGFAGPYALSSKVLKSLYKPSLSRRDRNNIRKLGALSRLLRSHE------NWSELH--KEFLTSQLLLCQEEVRHACKFGIPK-------NVSA-KSSMVWGKEAVSYVL 1599
VSV             WLFSDVL--SIDFIGPFSISTTLLQILYKPFLSGKDKNELRELANLSSLLRSGE------GWEDIH--VKFFTKDILLCPEEIRHACKFGIAK-------DNNKDMSYPPWGRESRGTIT 1610
PFRV            WIFSDVR--TKKFLGPFSLSTDALRCLYKSKLSKKDKNAVRNLSQLSSRMRTG-------DLSDEE--IGKIEARFSFTPAEMRHACKFTIGR-------SSVP-NIMSNWGPEAYGSIT 1595
SMRV            VIFADMR--SERIMGPLILSSMAMKHLFKSNITPADTEKLRLIANLSSALRDE-------DCPTPD--LSSLSSKCYTVDSEVRHACKFFIGK-------QASG-AKTLKWGQEVTGTIV 1609
BEFV            WVFSDMR--TNEVICSFGLSHLTYRILIQEGLNKDMKLRLQQCQDLYINIMTESKEELDKSSAKRE--IVECLRELKFVSSEIRHAVKFRYITGEKGEVQLIKEMEERRTWGDEYTGKAN 1652
EBLV-1          WIFSDFRSIKMTYLTLITYQSHLLLQRIDRNLSKQMRVRLRQLNSLMRQVLGGHGEGTLDSDEDIQGLLRDALQRTRWVDQEVRHAAKTMKCD------YSPSKRVSRKAGCSEWICSAQ 1640
KHUV            WIFSDFRSTKMTYLTLVTYQSHILLQKIEKSLSKQMRIRLRQLSSLMRQVLGGHGEDSLDSDEDFQGLLRDALQRTRWVDQEVRHAAKTMTGD------HSPTKKVSRKAGGSEWICSAQ 1640
RABV            WIFSDFRSVKMTYLTLITYQSHLLLQRVERNLSKSMRATLRQMSSLMRQVLGGHGEDTLESNDDIQRLLKDSLRKTRWVDQEVRHAARTMNGD------YSPDKKVSHKAGCSEWVCSAQ 1640
                 :*:*.    .          .         .  :.      ::    :   :            .                  *:*** :                     *

CIN6514         SIPVDYSSQSV--PKNLKP---CPRIQNPSISGFRLGQLPTGAHYKLRSILRKKNIHYRDALCGGDGSGGMTAAVLRYNLKARAIFNSILDFDGSTMKGASPDPPSALETVV-NGRTRCV 1713
CIN0327         SIPVDYSSQSV--PKNLKP---CPRIQNPSISGFRLGQLPTGAHYKLRSILRKKNIHYRDALCGGDGSGGMTAAVLRYNLKARAIFNSILDFDGSTMKGASPDPPSALETVV-NGRTRCV 1713
CIN0728         SIPVDYSSQSV--PKNLKP---CPRIQNPSISGFRLGQLPTGAHYKLRSILRKKNIHYRDALCGGDGSGGMTAAVLRYNLKARAIFNSILDFDGSTMKGASPDPPSALETVV-NGRTRCV 1713
ISFV            DIPVEFTSQKQ--TKHLNA---CPRIQDPTISGLRLGQLPTGAHYKIRTILNAYNIKCRDVLCGGDGSGGMTAACLRYYSNSRAIFNSILEFDGSSMKGSSPDPPSALETVD-QGMVRCV 1713
VSV             TIPVYYTTTPY--PKMLEM---PPRIQNPLLSGIRLGQLPTGAHYKIRSILHGMGIHYRDFLSCGDGSGGMTAALLRENVHSRGIFNSLLELSGSVMRGASPEPPSALETLG-GDKSRCV 1724
PFRV            SYPVFYSTGPT--TKPDWS---FSRLQNPTVSGLRVSQQATGAHYKLRSILKGLKIHYQDAIGCGDGSGGLSSCLLRENKHCKVIFNSLLELTGNTLRGSTPDPPSAINGIP-QIRGRCV 1709
SMRV            QVKVMFSDKPSKASKLSEV---APRVQNPTISGLRQVQLATGSHYKIRSIIKEMGIKYKDFISGGDGSGGITAACLRLNQGSRCIFNSLLEYDQVTLKGSAPSPPSAVVAMG-GMSERCV 1725
BEFV            LLDVWYLTSQSSENKNLVKGIKIPYHSNPTISGLRINQIATGAHYKLRTLINMTRITYRDFICGGDGSGGMTSCLLRLKPLSRGVFNSLLILDDKPLHGTRPSPPTAIMELG-EDSLRCV 1771
EBLV-1          QVAVSTSSNPAP-ISEIDIRALSKKLQNPLISGLRVVQWATGAHYKLKPILNDLEAYPTLCLVVGDGSGGISRAVLSMFPDAKLVFNSLLEVNDLMASGTHPLPPSALMSGGEDIVSRVI 1759
KHUV            QVAISTSSNPAP-TSELDVRALSRKFQNPLISGLRVVQWATGAHYKLKPILDDLESCPSLCLVVGDGSGGISRTVLSMFPDARLVFNSLLEVSDLMASGTHPLPPSAIMSGGDDIVSRVI 1759
RABV            QIAVSTSANPAP-VSELDIRALSKRFQNPLISGLRVVQWATGAHYKLKPILDDLNVFPSLCLVIGDGSGGISRAVLNMFPDSKLVFNSLLEVNDLMASGTHPLPPSAIMSGGDDIISRVI 1759
                   :           .               .:* :**:*  * .**:***::.::            :  ******::    *      .: :***:*            *: * **:*:           * :
          VI
CIN6514         NAESCWENPSDLSEQRTWDYFKFLKTHHGLKIDLIVLDMEVRDFAISASIEKCVRHNVSSILEEDGVLIYKTYGSTIAAESSNAVVNIGVLFESVELIQTEYSSTSTSEVYMYCRKIKKF 1833
CIN0327         NAESCWENPSDLSEQRTWDYFKFLKTHHGLKIDLIVLDMEVRDFAISASIEKCVRNNVSSILEEDGVLIYKTYGSTIAAESSNAVVNIGVLFESVELIQTEYSSTSTSEVYMYCRKIKRF 1833
CIN0728         NAESCWENPSDLSEQRTRDYFKFLKTHHGLKIDLIVLDMEVRDFAISASIEKCVRNNVSSILEEDGVLIYKTYGSTIAAESSNAVVNIGVLFESVELIQTEYSSTSTSEVYMYCRKIKKF 1833
ISFV            NATTCWENPSDLSQERTWDYFLHLKKSFNMKIDLIILDMEVRDFQISKLIEGNLRLKISKLLEKNGTLIYKTYGTIICSETSNVLTTLGPLFHSVYIVQTGYSSSFTSEVYVLFSKQKSF 1833
VSV             NGETCWEYPSDLCDPRTWDYFLRLKAGLGLQIDLIVMDMEVRDSSTSLKIETNVRNYVHRILDEQGVLIYKTYGTYICESEKNAVTILGPMFKTVDLVQTEFSSSQTSEVYMVCKGLKKL 1844
PFRV            NLDNVWEYPSDLSHPDTWKYFKDLKEQFNLDIDLIVMDMEVQDVTISRKIEQNLRDSVASLLSKQGTVIYKTYMTILSENDQSVLETVGVLFEDVQLCQTQYSSSQTSEVYCVMRRLRQR 1829
SMRV            NLEYCWQTPSDLSQEATWDNFRKEKDDHNLSLGLIVLDMEVRDHSTASRIAQLTRDYAYLLMAPHGTLIFKTYLTVLGTFEDNPLSILAPIFSDVRLVQSQFSSSHTSEVYLVGRRSRGG 1845
BEFV            NCYDVWKEPSDLSKQETWKYFVKLKKQNSMMIDLIVLDMEIINDEVIEDIYQNTKNHLIYLLEEGGCLIIKTYLTYLLKENTNILDMLGHLFTSVQLINTNLSSMKTSEIYVLFKNYKNR 1891
EBLV-1          DFNSIWEKPSDLRNPSTWRYFQTVQSAANMSYDLIICDAEVTDIPSINKITLLMSDFSLSING-PLSLIFKTYGTMLVNPDYKAVQHLSRAFPSVTGYITQMTSSFSSELYLKFSKRGKF 1878
KHUV            DFESIWEKPSDLRNLSTWRYFQSVQNSSKMSYDLIICDAEVTDISSVNKITLLMSDFSLSING-PLTLIFKTYGTMLVNSDYKAIQHLSRAFPSVTGYITQMTSSFSSELYLKFSKRGKF 1878
RABV            DFDSIWEKPSDLRNSATWRYFQSVQKQVNMSYDLIICDAEVTDIASINRITLLMSDFALSIDG-PLYLVFKTYGTMLVNPDYKAIQHLSRAFPSVTGFVTQVTSSFSSELYLRFSKRGKF 1878
                :    *: **** .  *    *    :     :  .**: * *: :        *           :       :: *** : :     . :  :.  *   *     :  :*   :**:*
```

```
CIN6514         VDAPH-PDWISLDYY--WSKLLCFRSYKEEFFRSYEVSRKESLKGIPNSFIPDPLVNLETLLQIAGVPSGISHQLAIDIKESQ-LTQITA-AMVLCGMIANYTLDVTKK-----RDSYNP 1943
CIN0327         VDAPH-PDWISLDYY--WSKLFCFRSYKEEFFRSYEVSRKESLKGIPNSFIPDPLVNLETLLQIAGVPSGISHQLAIDIKESQ-LTQITA-AMVLCGMIANYTLDVTKK-----RDSYNP 1943
CIN0728         VDAPH-PDWISLDYY--WSKLFCFRSYKEEFFRSYEVSRKESLKGIPNSFIPDPLVNLETLLQIAGVPSGISHQLAIDIKESQ-LTQITA-AMVLCGMIANYTLDVTKK-----RDLYNP 1943
ISFV            VDSPY-VDWGSLQYN--WEKLACFRNPRQEFKRALRIRSSRSLMGIPSSFLPDPLVNLETLLQISGVPSGVSHQLVTDVKSSG-ASGLSS-AIGLLGLISHFTLDVTKLY----VQEYRP 1944
VSV             IDEPN-PDWSSINES--WKNLYAFQSSEQEFARAKKVSTYFTLTGIPSQFIPDPFVNIETMLQIFGVPTGVSHAAALKSSDRP-ADLLTI-SLFYMAIISYYNINHIRVG----PIPPNP 1955
PFRV            VDSKF-VDWQSLVRQGIGSKIYCNQSLENEFNRALRIYQVDTLVGVPKELVPNLAVELETLLEIGGLSGGILGKLVLDLEEGR-LGFTMG-LIVSCILISEAAINTTRL-----ALKKEY 1941
SMRV            VMIPASIQWDVLNLA--VKKMACWRTDKEEFDRALSVSRLNHFKGIPKTLIPDVGSDLTVLMQAAGLEGGVATMVSHLLTHSP-SGLKGSDVMTILAVTANAVVNITRAT----LTRPGP 1958
BEFV            LTPCLQFDRNIILDN--WSFFYINKPIREEFERARELLNEDLGMGMPKELEPNPLIELSQMLQNSGVDGVAMSAISQEENLSF-FSTKELAVICLIIISESHLVTTKKHKNDCNCLQKKP 2008
EBLV-1          FRDSEYLTSSTLREMS--LVLFNCSSSKSEMLRARSLNYQDLVRGFPEEIISNPYNEMIITLIDNDVESFLVHKMVDDLELRQGAFSKMSIILTIMMVFSNRVFNVSKPLN---DPKFYP 1993
KHUV            FRDAEYLTSSTLREMS--LVLFNCSSPKSEMQRARSLNYQDLVRGFPEEIISNPYNEMIITLIDSEVESFLVHKMVDDLELQKGALSKMSIIIAIVMVFSNRVFNVSKPLS---DPMFYP 1993
RABV            FRDAEYLTSSTLREMS--LVLFNCSSPKSEMQRARSLNYQDLVRGFPEEIISNPYNEMIITLIDNDVESFLVHKMVDDLELQRGTLSKVAIIISIMIVFSNRVFNISKPLT---DPLFYP 1993
                .          :        :      ..*: *:  :       *.*. : .:   ::   :    :                         :    :     .   :

CIN6514         PSDGRLIRMSAALVGISFWISVKYYDKELNFELEQILSRSFP--IRWMLSHNYLFWTTK-----KGFRN---AKDVRLSGNMANIGNWIRCMELLHLPPGS-----LSKDEVTTTCGKYI 2048
CIN0327         PSDGRLIRMSAALVGISFWISVKYYDKELNFELEQILSRSFP--IRWMLSRNYLFWTTK-----KGFRN---AKDVRLSGNMANIGNWIRCMELLHLPPGS-----LSKDEVTTTCGKYI 2048
CIN0728         PSDGRLIRMSAALVGISFWISVKYYDKELNFELEQILSRSFP--IRWMLSHNYLFWTTK-----KGFRN---AKDVRLSGNMANIGNWIRCMELLHLPPGS-----LSKDEVTTTCGKYI 2048
ISFV            PSDNRLIKMASAITGISYWISIAYHDQQLNQALTSVIKKSFP--IRWGLINHRLHWSVS-----DRFHR---SKDVRLSDCLAGIGNWIRGMELMKLPAGM-----FSHKEVNMILSKYI 2049
VSV             PSDGIAQNVGIAITGISFWLSLMEKDIPLYQQCLAVIQQSFP--IRWEAVSVKGGYKQK-----WSTRGDGLPKDTRTSDSLAPIGNWIRSLELVRNQVRLN---PFNEILFNQLCRTVD 2065
PFRV            PSSGSCQRLAVCLVGAAVLLSVQHKSVENHKGVIRMLRTSVP--IRITHQSGKEGKIKARW---SSVSRTGLAKDVRLTSNMAGVGAWIRVWSRMKHFERK-----WEAQEADHWLKLHN 2051
SMRV            PSDQNCVSLASMITGSLIWISIWSGDFKLNQFANLLISTTFP--LFWNTWEVKDKKGNKLWKEGWSVKKGLINKSSRLDAKMANVGHWIRTWSRLNWVPEP----TFNAQDLDHKLSYFN 2072
BEFV            YSDQEIKSWMSGIIGIGLYLSLLDNKTNSFEILDYLINSDFKS-IHVNFNKERELCWSINYSSNEKKGKDLWKKRFSVKDKMAFMGNWIRLLHRQKVKNQKC---EYKEGRINNFLKFIN 2124
EBLV-1          PSDPKILRHFNICCGTLIYLSAALGDVLNFARLHELYNNPVTYYFKKQTLGGRMYLAWS------WTDNTPVFKRVACNSSLSLSSHWIRLIYKIVKTTRLVGSAKDLSQEVEKHLKSYN 2107
KHUV            PSDPKILRHFNICCSTLMYLSTALGDVLNFARLHELYNNPVTYYFKKQVIRGSIYLSWS------WSDSTSVFKRVACNSNLSLSSHWIRLIYKIVRTTRFVGSSSDLSEEVEKHLRGYN 2107
RABV            PSDPKILRHFNICCSTMMYLSTALGDVPNFARLHDLYNRPITCYFRKQVIRGNIYLSWS------WSDDTPVFKRVACNSSLSLSSHWIRLIYKIVKTTRLIGSIKDLSGEVERHLHGYN 2107
                 *.           .    :*    .         :    .   :                            *       ::  . ***

CIN6514         RNLKYSVILQQTGIIDLWKSRVASDDRSLMEVKTEFIESEHWVD 2092
CIN0327         RSLKYSVILQQTGIIDLWKSRVASDDRSLMEVKTEFIESEHWVD 2092
CIN0728         RSLKYSVILQQTGIIDLWKSRVASDDRSLMEVKTEFIESEHWVD 2092
ISFV            RGLNYHTISSRTGILEILKSQFSIIDRSLMTITTDNIQSSDWTD 2093
VSV             NHLKWSNLRRNTGMIEWINRRISKEDRSILMLKSDLHEENSWRD 2109
PFRV            QSLSISHAGRHTGVMTILHGAGDRLDRSVPTISSAPRDSGSWVE 2095
SMRV            IGLTATAVERQTGLLDLLSCQGTLASAEYHDVWSEVTPDMAWRD 2116
BEFV            KGLNLQKVKSQY-------------EEEIAKLL----------- 2144
EBLV-1          RWINFSDLRSRSSLLDYSCL------------------------ 2127
KHUV            RWIKFHDIRSRSSLLDYSCL------------------------ 2127
RABV            RWITLEDIRSRSSLLDYSCL------------------------ 2127
                  :.      .
```
