# Supplementary material for: Whole Genomes of Chandipura Virus Isolates and Comparative Analysis with Other Rhabdoviruses
Source: PLoS One. 2012 Jan 17;7(1):e30315. doi: 10.1371/journal.pone.0030315 (PMC3260278; doi:10.1371/journal.pone.0030315)
Supplement: Table S1 — (PDF) [file pone.0030315.s006.pdf]

Supplementary Table S1: Primers used for the amplification and sequencing of the CHPV whole genomes.

Name of the gene	Primer name	Sequence 5′-3′	Nucleotide position
**N and NS**	NF1	TATAGTAGTACACGAACACT	31-50
	NF2	TCTTTGGTCTTTATCGTGTGT	481-501
	NF3	TTGACCAAGCTGATTCCTACAT	871-892
	NF4	TAGGAGATATTCGAGTGAACT	1279-1299
	NF5	TGAGTGCTCTCCAACTTCTGCAGT	1742-1765
	NF6	CAGATTCTCTGTTGCTTACCACT	2281-2306
	NF7	TCAGTACAGGATGATTTGTT	825-844
	NF8	CTACTGAGATACCGGATCCTC	1571-1591
	NF9	TCAAAATGAGTTCTCAAGTATT	61-82
	NF10	CTACAGGTCAAACAGTCTCCGT	91-112
	NR1	TCTTCTTGTACTCGACCTGT	531-512
	NR2	TTGAAGAGTAAGGAGACTTCGT	942-921
	NR3	TCCTGGCGTACTCTGCAACT	1320-1301
	NR4	TGTGCTGATCTGCAACAGCCT	1830-1810
	NR5	TTCTTCAGAGCTTGCATCTTGAT	2331-2309
	NR6	TTCTCAATTGAGTCGAGAGTG	1436-1416
	NR7	TCTCCTCTGCATCCTTCGTGC	588-568
	NR8	GTTGAAGAGTAAGGAGACTTCG	943-922
**M**	MF1	TCAGAATCAGATGTCATCTT	34-53
	MF2	ATGCAACGTCTGAAGAAGTTTA	142-163
	MF3	GCTGACTACAGTGGATACATT	427-447
	MR1	TTCTACCACTCCGCGATCGAG	540-520
	MR2	TCAATGACTCTTAGAAATCAG	831-811
	MR3	CATAGGGCAGTATCTAGTAC	1020-1001
**G**	GF1	ATGACTTCTTCAGTGACAATTAGT	27-50
	GF2	GTCTTGTGGTTATGCTTCTGT	425-445
	GF3	TGTGTCCGACCGGGATCAGAGGT	853-875

**Name of the gene**	**Primer name**	**Sequence 5′-3′**	**Nucleotide position**
	GF4	GACAATGAACTACACGAGCT	1278-1297
	GR1	TCATCCACCGGGTTGAGATCCAT	1741-1708
	GR2	TGAGCATGAGGTAGCTGTGGAT	1342-1321
	GR3	TCCTCTGAATCTCTGAGGTC	930-911
	GR4	TGATTACCAAGAACTCAGAGT	471-451
	GR8(Stop))	TCATACTCTGGCTCTCATGTTGTT	1566-1543
	GR8	TCACCAGGAACTCGGAGTCAG	415-395
**L**	LF1	AACAGAGATA ATGGATCTCA	1-20
	LF2	TGGATGATGCTGCAGAGTTAT	27-47
	LF3	TCTTGGTCGCGTCATCTTTGATT	598-620
	LF4	CGTTGGTC GTCTACGGTT C	1023-1041
	LF5	TGGATTATCTGATGAGGATTTGGT	1527-1551
	LF6	TGGAGCATT CTTAATTTGC TGGT	2041-2064
	LF7	TTATCCATCATAGCACCAGTGAT	2717-2740
	LF8	TCAGAGTCTACCTCGATTCTT	3452-3469
	LF9	GTCTTATCATGTGGGTAGGAT	4054-4074
	LF10	TGCCTATACAAGCCTGCTTTATCT	4571-4594
	LF11	TTCACCAGATCCACCTAGT	5092-5110
	LF12	TCTCGAGACATTACTTCAGAT	5671-5691
	LF13	TATCCGGGAATATGGCT	6042-6058
	LF12-2	TAGACGAAGAGGCTAGTGAT	348-367
	LF13-2	GGAGATCACTGGCATGAGCT	1301-1320
	LF14	TATAATGAAAGACCGGATCT	1925-1944
	LF15	GATGCAATCTGCTGACTATCT	2281 -2301
	LF16	GTAGCGAGCAGCATTGTCTCT	3053-3073
	LF17	TGGATTATATTTCTGTCCATATTCCT	3367-3388
	LF18	CAT TCATTTCCATGTTGCCTGT	3856-3877
	LF19	GAGGATTATGGATTGATCT	4787-4805

Name of the gene	Primer name	Sequence 5′-3′	Nucleotide position
	LF20	GTATAGCAGTACATCCACTT	5452-5471
	LR3	TCACTGCTGTCATATCAT	1718-1701
	LR4	TTATTAGTTCCTGACTCGAT	2240-2231
	LR5	TTCGCAGCACTCATTCCCAT	2868-2848
	LR6	TAGTCTAGTAGCTCTTCTTAT	3526-3506
	LR7	TCTCATTTGAATGCTGAGT	4162-4144
	LR8	TTGCTGATCTTCCTTATATT	4629-4609
	LR9	TGTTCTCTGTTCACTCAGAT	52005181
	LR10	TGATCTGAGTCAGCTGTGACT	5762-5742
	LR11	TATGATTAATCTACCCAATGCTCAGATT	6294-6267
	LR12	TGATCT TATCGCCTGTTGAGT	803-783
	LR13	TCAGATCAGGTATGTCGAAGC	1355-1335
	LR14	CCACCTGCCTGTCCATTCCAGC	2016-1995
	LR15	TCTTAGTTCACTCTTGACGAT	3163-3143
	LR16	TCAACTGGAATGGACCAGATC	4821-4801
	LR17	TAGGTACCTTCCCATAATT	2321-2303
	LR18	TGGATGCACTTATCTTGTCT	4319-4300
	LR19	GGCTTGTCTCAACTCGATATCG	2188-2167
**Leader**	LeF1	ACGAAGACAAAAAAACCATTTAAACGATTATAT	
**Trailer**	TeR1	ACGAAGAAAACAAAACCAGTTATACGATTAAATTGTACA	
