# Supplementary material for: Whole Genomes of Chandipura Virus Isolates and Comparative Analysis with Other Rhabdoviruses
Source: PLoS One. 2012 Jan 17;7(1):e30315. doi: 10.1371/journal.pone.0030315 (PMC3260278; doi:10.1371/journal.pone.0030315)
Supplement: Table S2 — (PDF) [file pone.0030315.s007.pdf]

Supplementary Table S2: Predicted antigenic determinants (A) and conformational epitopes (B) in the G protein of CHPV.

(A)

AD No.	Antigenic determinant
1	1-YLS-3
2	51-PSKsD-55
3	71-RWYGPKYIT-79
4	85-IKpTRSDcDT-94
5	97-AsYKSGTLVSPG-108
6	111-PESCGYASVTDsEFL-125
7	149-VGgEcDQSYcDtIhN-163
8	170-ADQTKKNIcGQSFTPLT-186
9	192-DKtKeITAGG-201
10	224-GRN-226
11	242-TRIQE-246
12	249-LLpLfKEcPAGTEvR-263
13	271-AQVLtSEIQRILdYS-285
14	292-DKvERKEP-299
15	353-PsGISSD-359
16	388-WHlIGMGIVDNElHELS-404
17	420-IADDsE-425
18	440-ELvTGWFT-447
19	455-AGVVLILVVVLiY-467
20	473-FPvLCTtCRkPKWKK-487
21	494-SFEMRIFKPNNMRA-507

(B)

CE No	AD within 6A of Reference AD	Res within 6A of Ref AD
1	51: PSKsD :55, 170: ADQTKKNIcGQSFTPLT :186	48: G , 133: H
2	71: RWYGPKYIT :79, 111: PESCGYASVTDsEFL :125	-
3	85: IKpTRSDcDT :94, 97: AsYKSGTLVSPG :108, 149: VGgEcDQSYcDtIhN :163	-
4	97: AsYKSGTLVSPG :108, 85: IKpTRSDcDT :94 , 111:PESCGYASVTDsEFL :125 , 440: ELvTGWFT :447	-
5	111: PESCGYASVTDsEFL :125, 71: RWYGPKYIT :79 , 97:AsYKSGTLVSPG :108	-
6	149: VGgEcDQSYcDtIhN :163, 85: IKpTRSDcDT :94 , 455:AGVVLILVVVLiY :467	19: K , 20: N
7	170: ADQTKKNIcGQSFTPLT :186, 51: PSKsD :55 , 224: GRN :226 , 249: LLpLfKEcPAGTEvR :263	42: L , 47: I
8	192: DKtKeITAGG :201, 249: LLpLfKEcPAGTEvR :263	37: Q , 215: A
9	224: GRN :226, 70: ADQTKKNIcGQSFTPLT :186	-
10	242: TRIQE :246, 249: LLpLfKEcPAGTEvR :263	239: D
11	249: LLpLfKEcPAGTEvR :263, 170: ADQTKKNIcGQSFTPLT :186 , 192: DKtKeITAGG :201 , 242: TRIQE :246	215: A , 216: R , 219: R
12	292: DKvERKEP :299, 388: WHlIGMGIVDNElHELS :404 , 473: FPvLCTtCRkPKWKK :487	288: Q , 289: N
13	388: WHlIGMGIVDNElHELS :404, 292: DKvERKEP :299	303: L , 341: P , 342: V , 368: G
14	420: IADDsE :425, 494: SFEMRIFKPNNMRA :507	491: R
15	440: ELvTGWFT :447, 97: AsYKSGTLVSPG :108 , 420:IADDsE :425 , 473: FPvLCTtCRkPKWKK :487	436: K , 491: R
16	455: AGVVLILVVVLiY :467, 149: VGgEcDQSYcDtIhN :163	-
17	473: FPvLCTtCRkPKWKK :487, 292: DKvERKEP :299 , 440:ELvTGWFT :447 , 494: SFEMRIFKPNNMRA :507	436: K , 437: N
18	494: SFEMRIFKPNNMRA :507, 420: IADDsE :425 , 473:FPvLCTtCRkPKWKK :487	-
